# The Occurrence of Flavonoids and Related Compounds in *Cedrus brevifolia* A. Henry ex Elwes & A. Henry Needles. Inhibitory Potencies on Lipoxygenase, Linoleic Acid Lipid Peroxidation and Antioxidant Activity

**DOI:** 10.3390/plants7010001

**Published:** 2017-12-27

**Authors:** Andreas Douros, Dimitra Hadjipavlou-Litina, Konstantinos Nikolaou, Helen Skaltsa

**Affiliations:** 1 Department of Pharmacognosy & Chemistry of Natural Products, School of Pharmacy, National and Kapodistrian University of Athens, Panepistimiopolis, Zografou, 15771 Athens, Greece; andour@pharm.uoa.gr; 2 Department of Pharmaceutical Chemistry, School of Pharmacy, Aristotle University Thessaloniki, 54124 Thessaloniki, Greece; hadjipav@pharm.auth.gr; 3 Department of Forests, Ministry of Agriculture, Natural Resources and Environment, Nicosia 1414, Cyprus; knikolaou@fd.moa.gov.cy

**Keywords:** *C. brevifolia*, flavonoids, catechin, simple phenols, apocarotenoids, bioactivity, antioxidant, reducing power, total antioxidant capacity, reactive oxygen species

## Abstract

The phytochemical analysis of the polar extracts of *Cedrus brevifolia* needles yielded 20 compounds, namely from the methanol extract we isolated three flavonoids (**1**–**3**), one hydrolysable tannin (**4**), eleven phenolic derivatives (**5**–**15**) and one apocarotenoid (**16**), while from the methanol: water (5:1) extract we isolated four flavonoids (**17**–**20**). Chemical structures of all isolated compounds were determined by 1D, 2D-NMR (1 Dimension, 2 Dimensions Nuclear Magnetic Resonance) and UV-Vis (Ultraviolet-Visible) spectroscopy. Furthermore, the antioxidant potentials and the anti-inflammatory activities of both crude extracts and isolates were evaluated through DPPH radical scavenging capability, linoleic acid lipid peroxidation inhibition, and soybean LOX inhibition assays. This is the first report on the chemical profile of *C. brevifolia* needles. Catechin was the main compound derived from the methanol extract. According to our results, 4-*O*-β-d-glucopyranyl *trans*-p-coumaric acid and taxifolin were the most active ingredients.

## 1. Introduction

*Cedrus brevifolia* (Pinaceae) is an important endemic tree of Cyprus flora with narrow distribution. It is well-differentiated from other species of the genus based on morphological and eco-physiological traits, such as short needles and slow growth, resistance to aphids, and the highest tolerance to drought in all cedar species [1]. In ancient times, Theophrastus (371–287 B.C.) was the first to mention the existence of *Cedrus* in Cyprus, Phoenicia and Syria as an important forest tree of that period [2]. Cedar wood has been highly appreciated since ancient times for building temples, palaces, and ships [2,3]. The Roman author and architect Marcus Vitruvius Pollio wrote that the material used for the roof of the Greek temple of Artemis in Ephesus was from *Cedrus* wood [4]. In ancient Egypt, it was known that cedar was very resistant to insects and pathogenic microorganisms, so they used its essential oil to mummify corpses [3].

In South-West Turkey the tar extract from *C. libani*, under the common name *katran*, is used internally and externally to heal wounds, fight parasites, and cure various diseases [3]. It is noteworthy that the tar extract has been proposed to be recognized for its therapeutic value by the French pharmacopoeia [5]. *C. brevifolia* bark is a source of compounds with antioxidant capacity and 15-lipoxygenase inhibitory activity [6]; *C. deodara* needles water extract exhibits antibacterial activity [7].

Taking in consideration the importance and uses of *Cedrus* species, this study was designed to investigate the chemical composition of the methanol and the aqueous methanol [MeOH:H_2_O (5:1)] extracts prepared from needles of *C. brevifolia* and to evaluate their total antioxidant capacity and anti-inflammatory activity, as well as of the isolates.

## 2. Results

**Figure 1:**
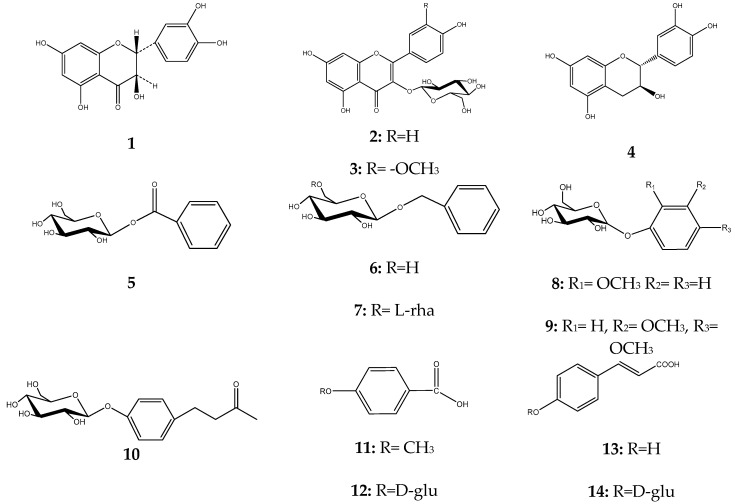

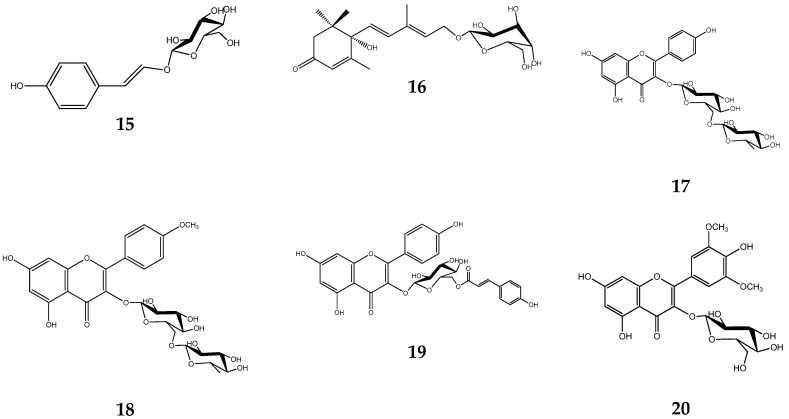
Structures of isolated compounds from *C. brevifolia* needles.

**Figure 2:**
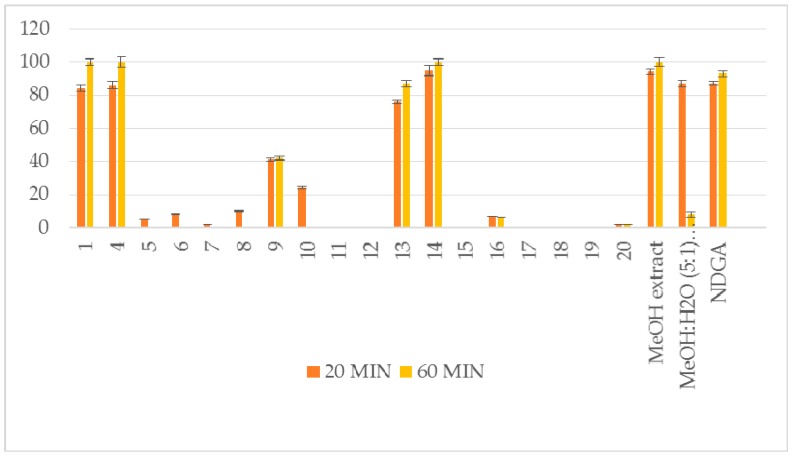
Reducing ability (RA %) at 0.1 mM. Interaction with DPPH.

**Figure 3:**
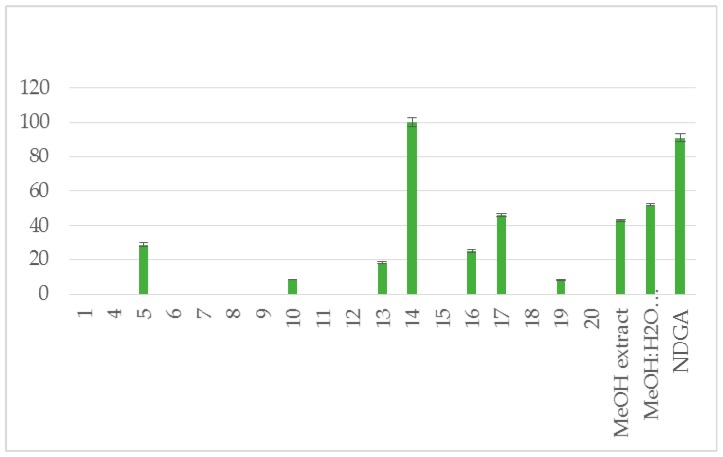
% Inhibition of soybean lipoxygenase (LOX) at 0.1 mM.

**Figure 4:**
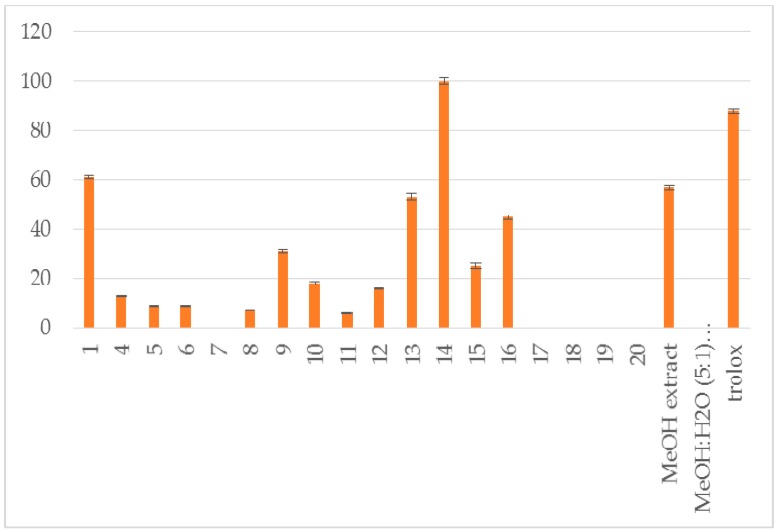
Percent inhibition of lipid peroxidation induced by AAPH at 0.1 mM.

The methanol extract (6.5 g) yielded taxifolin (**1**) [8], astragalin (**2**) [9], isorhamnetin 3-*O*-β-d-glucoside (**3**) [10], (−)-catechin (**4**) [11], benzoate glucoside (**5**) [12], benzyl-β-d-glucoside (**6**) [13], benzyl-β-d-rutinoside (**7**) [14], 2-methoxy-phenyl-β-d-glucoside (**8**) [15], 3,4-dimethoxyphenyl-β-d-glucoside (**9**) [16], raspberry ketone (**10**) [17], p-anisic acid (**11**) [18], 4-hydroxybenzoic acid 4-*O*-β-d-glucoside (**12**) [19], p-coumaric acid (**13**, 6.0 mg) and its glucoside (**14**) [20,21], *trans*-vaginoside (**15**) [22] and abscisic alcohol glucoside (**16**) [23]. The methanol:water (5:1) extract afforded kaempferol-3-*O*-β-rutinoside (**17**) [24], kaempferide-3-*O*-β-rutinoside (**18**) [25], tiliroside (**19**) [26], and syringetin 3-*O*-β-d-glucoside (**20**) [27] (Figure 1). Furthermore, both crude extracts and isolated compounds were examined for their inhibitory potency on lipoxygenase and lipid peroxidation, as well as for their antioxidant activity, in comparison to known antioxidants, e.g., caffeic acid, nor-dihydroguaretic acid (NDGA) and trolox. AAPH (2,2′-azobis (2-amidino-propane) dihydrochloride), DPPH (2,2-diphenyl-1-picrylhydrazyl) and soybean lipoxygenase (LOX) assays were used for the tests. This is the first report on the chemical profile of *C. brevifolia* needles. Catechin was the main compound derived from the methanol extract (See Supplementary Data, Table S1. According to our results of the in vitro tests, both extracts were found to possess potential antioxidant activity due to their high phenolic contents. Moreover, 4-*O*-β-d-glucopyranyl *trans*-p-coumaric acid and taxifolin were the most active ingredients (Figure 2, Figure 3 and Figure 4, Table 1).

## 3. Discussion

Overall, 20 compounds were isolated from *C. brevifolia* needles. The isolates were categorized as simple phenols, polyphenolic derivatives, and one apocarotenoid. Taking into account the phenolic nature of the isolates, we decided to evaluate their in vitro antioxidant activity using two different antioxidant assays: (a) interaction with the stable free radical DPPH, as this method can be used for polar and nonpolar constituents [28], (b) interaction with the water-soluble azo compound AAPH in order to measure the radical-scavenging activity in vitro [29]. The antioxidant ability of the isolates was measured in comparison to positive controls, such as NDGA and trolox. The results are shown in Table 1 and Figure 2, Figure 3 and Figure 4. The interaction, which indicates their radical scavenging ability in an iron-free system, was measured at 100μM after 20 and 60 min. In the DPPH assay, particularly effective antioxidants are the phenoxide anions from phenolic compounds like compounds **1**, **4**, **13** and **14**, as well as nor-dihydroguaretic acid (NDGA), which was used as a reference. For these compounds it was observed an increase in their antioxidant activity after 60 min. Methanol and methanol: water extracts presented high reducing activity. This could be correlated to the presence of phenolic derivatives. The rest isolates did not present any interesting result. Due to low amounts, compounds **2** and **3** were not tested.

The % inhibition of lipid peroxidation given in Table 1indicates three potent isolates, **1**, **13** and **16**. The methanol extract also exhibits anti lipid peroxidation activity.

We also decided to further evaluate the presented isolates and extracts for their ability to inhibit soybean LOX since most of the LOX inhibitors are antioxidants or free radical scavengers. Perusal of the % inhibition values (Table 1) shows that the most potent inhibitor is isolate **14** followed by **17** which seem to be less potent. It should to be noticed that both methanol extracts are almost equipotent. This inhibition is related to their antioxidant ability.

The investigation revealed that the polar extracts of *C. brevifolia* needles are abundant in phenolic compounds, which could explain its strong antioxidant activity.

## 4. Materials and Methods

### 4.1. Plant Material

*C. brevifolia* needles were collected on April 2013 from Cedar valley near Paphos (Cyprus) and authenticated by Mr. Konstantinos Nikolaou. A voucher specimen is kept at the Herbarium of Department of Forests, Cyprus, under the number: CYP 1467.

### 4.2. Equipment and Reagents

Optical rotation was recorded on a Perkin Elmer 341 polarimeter. The [α]_D_ values were obtained in methanol at 20 °C. UV spectra were recorded on a Shimadzu UV-160A spectrophotometer, according to [30] (1970). IR spectra were carried out by Perkin-Elmer Paragon 500 FT-IR spectrophotometer (PerkinElmer, Inc., Waltham, MA, USA). ^1^H, ^13^C and 2D-NMR spectra were recorded on a Bruker DRX 400 (Bruker BioSpin GmbH, Silberstetten, Germany), and on a Bruker AC 200 (50.3 MHz for ^13^C-NMR) spectrometers at 295 K. Chemical shift are reported in ppm (*δ*) using the residual solvent signal (δ_H_ 3.31 in ^1^H and δ_C_ 49.0 in ^13^C, CD_3_OD) as reference. Correlation spectroscopyΥ (COSY); Heteronuclear Single Quantum Correlation (HSQC); Heteronuclear Multiple Bond Correlation (HMBC); Nuclear Overhauser Effect Spectroscopy (NOESY); Rotating-frame Overhauser Effect Spectroscopy (ROESY) experiments were performed using standard Bruker microprograms. Vacuum liquid chromatography (VLC) was performed on a silica gel (Merck: 43–63 μm) (Merck KGaA, Darmstadt, Germany) [31], column chromatography (CC) on silica gel 60H (SDS: 40–63 μm), Cellulose (Merck, Art. 2330) (Merck KGaA, Darmstadt, Germany) and Sephadex LH 20 (Pharmacia, Sweden). Gradient elution with the solvents mixtures indicated in each case. Semi-preparative RP_18_-HPLC (Reversed Phase_18_-High Liquid Performance Chromatography) was performed on a HPLC system: PU-2080 pump Plus (JASCO, Tokyo, Japan); refractive index detector RID-10A (Shimazdu, Kyoto Japan); software: Clarity (JASCO, Tokyo, Japan), with Kromasil RP-18 columns (i.d.:10 mm, length: 250 mm, 10 µm); flow rate 1.5 mL/min. Prep. Thin Layer Chromatography plates pre coated with silica gel 60 (Merck, Art. 5721). Fractions monitoring to follow separation was performed by thin layer chromatography (TLC) on silica gel 60 F254 (Merck, Art. 5554) and cellulose (Merck Art. 5552). Compounds were detected using UV absorbance (λ 254 and λ 365 nm). Vanillin/sulphuric acid reagent (vanillin 5% in H_2_SO_4_/MeOH 1:1) and Neu’s reagent [32] were used for detection at TLC chromatography. Analytical solvents were obtained from Panreac Quimica SA (Barcelone, Spain, Italy), while deuterated solvents were purchased from Merck, KGaA (Darmstadt, Germany). Medium-pressure liquid chromatographic (MPLC) separations were carried out using Büchi C-615 system & pump Büchi 688 with reverse phase column packed with SiO_2_. Desiccators were activated by anhydrous di-phosphorus pentoxide analytical reagent (a.r.) grade (Art. CL 00. 0631; Chem-Lab, N.V., Belgium).

### 4.3. Equipment and Reagents for In Vitro Experiments

Soybean lipoxygenase, sodium linoleate, 2,2-azobis (2-amidinopropane) dihydrochloride (AAPH), 1,1-diphenyl-2-picrylhydrazyl (DPPH) were obtained from Sigma Chemical, Co. (St. Louis, MO, USA). For the in vitro tests, UV-Vis spectra were obtained on a 554 double beam spectrophotometer Perkin-Elmer (Perkin-Elmer Corporation Ltd., Lane Beaconsfield, Bucks, UK).

### 4.4. Extraction and Chromatography

*C. brevifolia* needles (0.143 kg) were extracted successively with dichloromethane, methanol, and methanol: water 5:1, and concentrated to dryness to yield residues of 23.0 g and 18.3 g, respectively.

10 g of the MeOH extract was submitted to RP_18_-MPLC (41.0 × 4.0 cm) using a H_2_O:MeOH gradient system (100% H_2_O→100% MeOH; steps of 10% MeOH) to yield 11 fractions (A-K) of 500 mL each. Based on TLC results, combined fractions D and E (149.6 mg) were further applied to CC over silica gel eluted with mixtures of DM: MeOH: H_2_O of increasing polarity (95:5:0.5 to 50:50:5) and gave 41 sub-fractions (D′A-D′L); subfraction D′L (6.0 mg) was identified as compound **13**; subfraction D′I (7.6 mg) was subjected to prep. TLC on silica gel with DM:MeOH:H_2_O 7:3:0.3 and afforded compound **8** (4.0 mg). Fraction H (236.7mg) was subjected to repeated CC over Sephadex LH-20 (MeOH 100%) and silica gel (DM: MeOH: H_2_O 95:5:0.5 to 50:50:5) and yielded compounds **11** (3.0 mg), **15** (2.8 mg). Fraction I (1.5 g) was submitted to CC over silica gel using mixtures of DM: MeOH:H_2_O (100:0:0 to 0:0:100) and 15 fractions were obtained. Subfraction IF (9.1 mg; eluted with DM: MeOH:H_2_O 80:20:2) was identified as compound **1** (9.1 mg); subfraction II (75.0 mg; eluted with DM: MeOH: H_2_O 70:30:3) was subjected to prep. RP_18_-HPLC (MeOH:AcOH 5% 4:6) and yielded compounds **2** (0.4 mg; Rt 40.4), **3** (0.5 mg; Rt 39.4), **5** (12.6 mg; Rt 14.8), **6** (3.8 mg; Rt 15.7), **9** (2.7 mg; Rt 9.39), **10** (8.4 mg; Rt 11.4), **12** (1.9 mg; Rt 43.6), **16** (3.4 mg; Rt 28.2); subfraction IJ (358.7 mg; eluted with DM: MeOH:H_2_O 70:30:3) was subjected to CC over silica gel (EtOAc:MeOH:H_2_O 100:0:0 to 70:30:3) and afforded compound **4** (19.0 mg); fraction IJK derived from the latter subfraction IJ (17.5 mg; eluted with EtOAc:MeOH:H_2_O 90:10:1) was further fractionated by RP_18_-HPLC (MeOH:AcOH 5% 4:6) and yielded compound **7** (2.8 mg; Rt 25.0); subfraction IL (259.6 mg) was submitted to CC over Cellulose (isocratic elution with AcOH:H2O15:85) and afforded compounds **4** (14.5 mg) and **14** (3.5 mg). Sub-fraction IK (142.0 mg) was subjected to CC over silica gel eluted with mixtures of increasing polarity of EtOAc/MeOH and yielded **4** (7.5 mg).

5.0 g of the methanol:water (5:1) extract also was submitted to RP_18_-MPLC (41.0 × 4.0 cm) using mixtures of decreasing polarity of H_2_O:MeOH (100:0 to 0:100; steps of 10%) and gave us 11 fractions (A-K). Fraction I (157.0 mg; eluted with H_2_O:MeOH 20:80) was subjected to CC over Sephadex LH-20 (MeOH 100%), and subfraction IF (70.5 mg) was further analyzed by RP_18_-HPLC (MeOH:AcOH 5% 4:6) and yielded compound **20** (0.8 mg; Rt 14.0). Subfraction IE (44.0 mg) submitted to CC over silica gel (DM:MeOH 95:5 to 0:100) and yielded compound **17** (1.0 mg; eluted with DM: MeOH 80:20). Fraction J (256.0 mg; eluted with H_2_O:MeOH 10:90) was applied to CC over silica gel (DM:MeOH: H_2_O 98:2:0.2 to 0:50:50); combined subfractions JK, JN (6.9 mg; eluted with DM:MeOH: H_2_O 97:3:0.2 to 95:5:0.3) and subfraction JR (8.2 mg; eluted DM:MeOH: H_2_O 85:15:0.6), were further fractionated by prep. TLC on silica gel (DM:MeOH:H_2_O 8:2:0.2) and afforded compounds **19** (5.7 mg) and **18** (2.8 mg), respectively. All obtained extracts, fractions and isolated compounds were evaporated to dryness in vacuum under low temperature, and then were put in activated desiccators with P_2_O_5_ until their weights had stabilized.

### 4.5. DPPH Radical Scavenging Activity

The reducing ability of *C. brevifolia* extracts and of the isolated compounds was determined using the method described by Pontiki et al. [33]. To an ethanolic solution of DPPH 1mL from an 100µM stock solution (freshly prepared), an equal volume of the extracts (stock solutions 5 mg/mL) and pure compounds (stock solutions 10mM) dissolved in EtOH, were added separately. The mixture was shaken vigorously and incubated at room temperature for 20 and 60 min. Absorbance was measured spectrophotometrically at 517 nm. NDGA was used as reference substance. All tests were performed in triplicate and the averages of the results were calculated.

### 4.6. AAPH Induced Linoleic Acid Lipid Peroxidation Assay

The anti-lipid peroxidation activity of *C. brevifolia* extracts and isolated compounds were determined, as reported previously [33], i.e. 10 μL of the 16 mM sodium linoleate was added to the UV cuvette containing 0.93 mL of 0.05 M phosphate buffer, pH 7.4, which was previously pre-thermostated at 37 °C. The oxidation reaction was initiated under air by the addition of 50 μL of 40 mM AAPH solution. Oxidation was carried out in the presence of samples (10 μL) without an antioxidant, and lipid peroxidation was calculated in the presence of same level of EtOH at 234 nm.

### 4.7. Soybean LOX Inhibition

LOX inhibition of the extracts and isolates were determined by using the method described by Pontiki et al. [33]. The samples, dissolved in EtOH, were incubated at room temperature with sodium linoleate (100 μL) and 200 μL enzyme solution (1/9 × 10^−4^ *w*/*v* in saline). The transformation of sodium linoleate to 13-hydroperoxylinoleate sodium was measured spectrophotometrically at 234 nm and compared with the appropriate reference NDGA (nor-dihydroguaiaretic acid).

### 4.8. Statistics

Experiments were performed in triplicate. The results were expressed as mean ± standard deviation (SD). When needed statistical comparisons were made using the Kruskal Wallis test. Statistically significant difference was defined as *p* < 0.05.

The reducing abilities are given only as % inhibition since the majority of the tested compounds presented lower than 50% antioxidant ability at 100 μM. Considering the LOX inhibition as well as the anti-lipid peroxidation activity, only one and two compounds respectively exhibited high activities at 100 μM. Thus for the sake of comparison we did not determine the IC_50_ values for them. The same concept was followed for the DPPH interaction results.

### 4.9. NMR Data of Compounds ***1**–**20***

The ^1^H- and ^13^C-NMR data of these compounds (**1**–**20**) are listed as follows (see also Supplementary Material):

*Compound* **1**: ^1^H-NMR (400 MHz, CD_4_O) δ_H_: 6.95 *d* (1H, H-2′, *J* = 1.8 Hz), 6.85 *dd* (1H, H-6′, *J* = 8.0, 1.8 Hz), 6.80 *d* (1H, H-5′, *J* = 8.0 Hz), 5.92 *d* (1H, H-8, *J* = 1.9 Hz), 5.88 *d* (1H, H-6, *J* = 1.9 Hz), 4.89 *d* (1H, H-2, *J* = 12.2 Hz), 4.50 *d* (1H, H-3, *J* = 12.2 Hz).

NOESY: nOe signals between H-2/H-3; H-3/H-6′.

*Compound* **2**: ^1^H-NMR (400 MHz, CD_4_O) δ_H_: 8.06 *d* (2H, H-2′, H-6′, *J* = 8.7 Hz) 6.90 *d* (2H, H-3′, H-5′, *J* = 8.7 Hz) 6.40 *s* (1H, H-8) 6.21 *s* (1H, H-6) 5.23 *d* (1H, H-1′′, *J* = 7.8 Hz) 3.42–3.20 *m* (3H, H-2′′,3′′,4′′,5′′), 3.69 *dd* (1H, H-6a′′, *J* = 12.0, 5.8 Hz) 3.53 *dd* (1H, H-6b′′ *J* = 12.0, 2.1 Hz).

^13^C-NMR (50.3 MHz CD_4_O) δ_C_: 161.0 (C-2) 100.0 (C-6) 94.4 (C-8) 158.4 (C-9) 104.0 (C-10) 123.0 (C-1′) 131.9 (C-2′,6′) 159.2 (C-4′) 115.1 (C-3′,5′) 103.7 (C-1′′) 76.2 (C-2′′) 78.1^a^ (C-3′′) 71.3 (C-4′′) 78.4^a^ (C-5′′) 62.3 (C-6′′).

^a^: interchangeable signals.

*Compound* **3**: ^1^H-NMR (400 MHz, CD_4_O) δ_H_: 7.93 *d* (1H, H-2′, *J* = 2.0 Hz) 7.59 *dd* (1H, H-6′, *J* = 8.6, 2.0 Hz) 6.89 *d* (1H, H-5′, *J* = 2.0 Hz) 6.19 *d* (1H, H-6, *J* = 2.0 Hz) 6.38 *d* (1H, H-8, *J* = 2.0 Hz) 3.95 *s* (3H, OCH_3_) 5.40 *d* (1H, H-1′′, *J* = 7.8 Hz) 3.40–3.20 *m* (3H, H-2′′,3′′,4′′,5′′) 3.22 *dd* (1H, H-5′′, *J* = 4.8, 3.0) 3.73 *dd* (1H, H-6a′′, *J* = 12.0, 5.3 Hz) 3.52 *dd* (1H, H-6b′′, *J* = 12.0, 1.8 Hz).

^13^C-NMR (50.3 MHz CD_4_O) δ_C_: 159.0 (C-2) 100.4 (C-6) 95.2 (C-8) 157.3 (C-9) 104.0 (C-10) 121.0 (C-1′) 114.7 (C-2′) 151.3 (3′) 149.7 (C-4′) 116.4 (C-5′) 103.7 (C-1′′) 123.8 (C-6′) 103.7 (C-1′′) 75.5 (C-2′′) 78.0 (C-3′′) 71.6 (C-4′′) 78.2 (C-5′′) 62.8 (C-6′′).

*Compound* **4**: ^1^H-NMR (400 MHz, CD_4_O) δ_H_: 6.82 *d* (1H, H-2′, *J* = 1.9 Hz) 6.74 *d* (1H, H-5′, *J* = 8.0 Hz) 6.71 *dd* (1H, H-6′ *J* = 8.0, 1.9 Hz) 5.82 *d* (1H, H-6, *J* = 2.2 Hz) 5.94 *d* (1H, H-8, *J* = 2.2 Hz) 4.56 (1H, H-2, *J* = 7.7 Hz) 3.97 *ddq* (1H, H-3, *J* = 8.1, 7.7, 5.4 Hz) 2.84 *dd* (1H, H-4ax, *J* = 16.1, 5.4 Hz) 2.50 *dd* (1H, H-4eq, *J* = 16.1, 8.1 Hz).

^13^C-NMR (50.3 MHz CD_4_O) δ_C_: 82.8 (C-2) 68.8(C-3) 28.4 (C-4) 157.6 (C-5) 95.5 (C-6) 157.8 (C-7) 96.3 (C-8) 156.9 (C-9) 100.8 (C-10) 132.4 (C-1′) 115.3 (C-2′) 146.4 (3′,4′) 116.0 (C-5′) 119.9 (C-6′).

*Compound* **5**: ^1^H-NMR (400 MHz, CD_4_O) δ_H_: 8.10 br *d* (2H, H-2,6, *J* = 7.7 Hz) 7.50 br *t* (1H, H-3,5, *J* = 7.7 Hz) 7.63 (1H, H-4, *J* = 7.7 Hz). 5.73 *d* (1H, H-1′, *J* = 7.6 Hz) 3.52 *dd* (1H, H-2′, *J* = 7.6, *Hz) 3.49–3.43 *m* (3H, H-3′,4′,5′), 3.86 *dd* (1H, H-6a,′ *J* = 12.1, 2.1 Hz) 3.71 *dd* (1H, H-6b′, *J* = 12.1, 5.1 Hz).

^13^C-NMR (50.3 MHz CD_4_O) δ_C_: 133.2 (C-1) 130.7 (C-2,6) 128.4(C-3,5) 132.4 (C-4) 166.2 (C-7) 94.1 (C-1′) 73.4 (C-2′) 77.8^a^ (C-3′) 71.3 (C-4′) 78.4^a^ (C-5′) 62.1 (C-6′).

*: partially overlapped signal; ^a^: interchangeable signals.

*Compound* **6**: ^1^H-NMR (400 MHz, CD_4_O) δ_H_: 7.42 br *d* (2H, H-2,6, *J* = 7.3 Hz) 7.33 br *dd* (2H, H-3,5, *J* = 7.7, 7.3 Hz) 7.28 br *d* (1H, H-4, *J* = 7.3 Hz) 4.95 *d* (1H, H-7a, *J* = 11.8) 4.69 *d* (1H, H-7b, *J* = 11.8 Hz). 4.38 *d* (1H,H-1′, *J* = 7.4 Hz) 3.43-3.31 *m* (3H, H-2′,3′,4′,5′) 3.91 *dd* (1H, H-6a′, *J* = 2.1, 1.7 Hz) 3.70 *dd* (1H, H-6b′, *J* = 11.7, 5.8 Hz).

^13^C-NMR (50.3 MHz CD_4_O) δ_C_: 138.9 (C-1) 128.9 (C-2,6) 128.9 (C-3,5) 128.4 (C-4) 71.4 (C-7) 103.0 (C-1′) 74.9 (C-2′) 78.0^a^ (C-3′) 71.6 (C-4′) 77.9^a^ (C-5′) 62.4 (C-6′).

^a^: interchangeable signals.

*Compound* **7**: ^1^H-NMR (400 MHz, CD_4_O) δ_H_: 7.42 br *d* (2H, H-2,6, *J* = 7.3 Hz) 7.33 br *dd* (2H, H-3,5, *J* = 7.7, 7.3 Hz) 7.28 br *d* (1H, H-4, *J* = 7.3 Hz) 4.87 *d* (1H, H-7a, *J* = 11.8) 4.64 *d* (1H, H-7b, *J* = 11.8 Hz) 4.32 *d* (1H,H-1′, *J* = 7.9 Hz) 3.24 *dd* (1H, H-2′, *J* = 8.5, 7.9) 3.70-3.20 m* (3H, H-3′,4′,5′), 3.99 *dd* (1H, H-6a′, *J* = 11.7, 1.7 Hz) 3.64 *dd* (1H, H-6b′, *J* = 11.3, 5.9 Hz) 4.81 *d* (1H,H-1′′, *J* = 1.7 Hz) 3.86 *dd* (1H, H-2′′ *J* = 3.1, 1.7 Hz) 3.68 *m* (3H, H-3′′, *J* = 9.5 3.1 Hz), 3.34 * (1H H-4′′) 3.67 (1H H-5′′) 1.27 *d* (3H H-6′′, *J* = 6.2Hz).

^13^C-NMR (50.3 MHz CD_4_O) δ_C_: 128.8 (C-2,6) 128.8 (C-3,5) 128.3 (C-4) 71.4 (C-7) 103.0 (C-1′) 75.0 (C-2′) 77.9^a^ (C-3′) 71.3 (C-4′) 77.4^a^ (C-5′) 67.6 (C-6′) 102.1 (C-1′′) 72.2(C-2′′) 71.1 (C-3′′) 73.7 (C-4′′) 71.1 (C-5′′) 17.9 (C-6′′).

*: partially overlapped signals; ^a^: interchangeable signals.

*Compound* **8**: ^1^H-NMR (400 MHz, CD_4_O) δ_H_: 7.14 br *d* (1H, H-3, *J* = 8.2 Hz) 7.09-7.07 *m* (2H, H-4,5) 6.92 *dd* (1H, H-6, *J* = 8.2, 1.8 Hz). 4.89* (1H,H-1′) 3.50 (1H, H-2′) 3.82 (-OCH_3_) 3.48 *m* (1H, H-3′) 3.40 (1H, H-4′) 3.91 *dd* (1H, H-6a′, *J* = 11.7, 1.7 Hz) 3.70 *dd* (1H, H-6b′, *J* = 11.7, 5.8 Hz).

^13^C-NMR (50.3 MHz CD_4_O) δ_C_: 117.0^a^ (C-3) 112.0 (C-4) 116.8 ^a^ (C-5) 120.2 (C-6) –OCH_3_ (56.1) 102.2 (C-1′) 74.5 (C-2′) 77.5 (C-3′) 71.0 (C-4′) 78.0 (C-5′) 62.0 (C-6′).

*: partially overlapped by methanol-d_4_ moisture; ^a^: interchangeable signals.

*Compound* **9**: ^1^H-NMR (400 MHz, CD_4_O) δ_H_: 6.83 *d* (1H, H-2, *J* = 2.8 Hz) 6.86 *d* (2H, H-5, *J* = 8.8 Hz) 6.67 *dd* (1H, H-6, *J* = 8.8, 2.9 Hz) 3.82 (-OCH_3_-3), 3.79 (-OCH_3_-4). 4.79 *d* (1H, H-1′, *J* = 7.5 Hz) 3.46–3.35 *m* (4H, H-2′,3′,4′,5′) 3.91 *dd* (1H, H-6a′, *J* = 11.7, 1.7 Hz) 3.70 (1H, H-6b′, *J* = 11.7, 5.8 Hz).

^13^C-NMR (50.3 MHz CD_4_O) δ_C_: 153.9 (C-1) 103.7 (C-2) 145.7 (C-3) 150.6 (C-4) 113.5 (C-5) 109.0 (C-6) –OCH_3_ (56.1, 57.5) 103.1 (C-1′) 74.9 (C-2′) 77.4^a^ (C-3′) 71.3 (C-4′) 77.9^a^ (C-5′) 62.2 (C-6′).

^a^: interchangeable signals.

*Compound* **10**: ^1^H-NMR (400 MHz, CD_4_O) δ_H_: 7.11 *d* (2H, H-3,5, *J* = 8.5 Hz) 7.00 *d* (2H, H-2,6, *J* = 8.5 Hz) 2.80–2.76 *m* (2H, H-7,8) 2.10 *s* (–CH_3_). 4.85 *d* (1H,H-1′, *J* = 7.8 Hz) 3.43 (1H,H-2′) 3.40–3.30 (2H, H-3′,4′,5′) 3.88 *dd* (1H, H-6a′, *J* = 12.0, 1.8 Hz) 3.69 (1H, H-6b′, *J* = 12.0, 5.0 Hz).

^13^C-NMR (50.3 MHz CD_4_O) δ_C_: 157.3 (C-1) 117.5 (C-2,6) 129.0 (C-3,5) 135.3 (C-4) 29.68 (C-7) 45.6 (C-8) 29.63(C-9 –CH_3_) 210.3 (>C = 0) 102.0 (C-1′) 74.6 (C-2′) 77.8 (C-3′) 71.0 (C-4′) 77.7 (C-5′) 62.0 (C-6′).

*Compound* **11**: ^1^H-NMR (400 MHz, CD_4_O) δ_H_: 7.96 *d* (2H, H-2,6, *J* = 7.89 Hz).

7.01 *d* (2H, H-3,5, *J* = 7.04 Hz) 3.88 *s* (-OCH_3_).

^13^C-NMR (50.3 MHz CD_4_O) δ_C_: 131.7 (C-2,6) 116.0 (C-3,5) 56.0 (–OCH_3_).

*Compound* **12**: ^1^H-NMR (400 MHz, CD_4_O) δ_H_: 8.06 *d* (2H, H-2,6, *J* = 8.1 Hz) 6.90 *d* (2H, H-3,5, *J* = 8.1 Hz). 4.96 *d* (1H,H-1′, *J* = 7.4 Hz) 3.50–3.30 *m* (4H, H-2′, 3′, 4′, 5′) 3.91 *dd* (1H, H-6a′, *J* = 11.7, 1.7 Hz) 3.70 (1H, H-6b′, *J* = 11.7, 5.8 Hz).

^13^C-NMR (50.3 MHz CD_4_O) δ_C_: 132.0 (C-2,6) 116.2 (C-3,5) 101.0 (C-1′) 75.0 (C-2′) 78.0 (C-3′) 71.8 (C-4′) 78.0 (C-5′) 62.3 (C-6′).

*Compound* **13**: ^1^H-NMR (400 MHz, CD_4_O) δ_H_: 7.44 *d* (2H, H-2,6, *J* = 8.3 Hz) 6.79 *d* (2H, H-3,5, *J* = 8.3 Hz) 7.56 *d* (1H, H-7, *J* = 16.0 Hz) 6.29 *d* (1H, H-8, *J* = 16.0 Hz).

^13^C-NMR (200 MHz CD_4_O) δ_C_: 131.4 (C-2,6) 124.3 (C-3,5) 146.7 (C-7) 115.4 (C-8).

*Compound* **14**: ^1^H-NMR (400 MHz, CD_4_O) δ_H_: 7.54 *d* (2H, H-2,6, *J* = 7.9 Hz) 7.12 *dd* (2H, H-3,5, *J* = 7.9 Hz) 7.60 *d* (1H, H-7, *J* = 16.2 Hz) 6.37 *d* (1H, H-8, *J* = 16.2 Hz). 4.96 *d* (1H, H-1′, *J* = 7.2 Hz) 3.70–3.41 *m* (4H, H-2′, 3′, 4′, 5′) 3.88 *dd* (1H, H-6a′, *J* = 12.1, 2.1) 3.70 (1H, H-6b′, *J* = 12.1, 8.1 Hz).

^13^C-NMR (50.3 MHz CD_4_O) δ_C_: 129.2 (C-1) 132.0 (C-2,6) 117.4 (C-3,5) 159.5 (C-4) 144.7 (C-7) 117.8 (C-8) 170.3 (C-9 –COOH) 101.2 (C-1′) 74.4 (C-2′) 77.5 (C-3′) 70.7 (C-4′) 78.0 (C-5′) 62.2 (C-6′).

*Compound* **15**: ^1^H-NMR (400 MHz, CD_4_O) δ_H_: 7.48 *d* (2H, H-2,6, *J* = 8.2 Hz) 7.08 *d* (2H, H-3,5, *J* = 8.2 Hz) 7.37 *d* (1H, H-7, *J* = 16.0 Hz) 6.40 *d* (1H, H-8, *J* = 16.0 Hz). 4.94 *d* (1H, H-1′) 3.55–3.28 *m* (4H, H-2′,3′,4′,5′) 3.89 *dd* (1H, H-6a′, *J* = 11.8, 2.1) 3.70 (1H, H-6b′, *J* = 11.8, 4.4 Hz).

^13^C-NMR (50.3 MHz CD_4_O) δ_C_: 129.4 (C-2, 6) 117.6 (C-3, 5) 139.9 (C-4) 129.9 (C-7) 102.2 (C-1′) 74.3 (C-2′) 77.6 (C-3′) 70.8 (C-4′) 76.5 (C-5′) 62.1 (C-6′).

*Compound* **16**: ^1^H-NMR (400 MHz, CD_4_O) δ_H_: 2.45 *d* (1H, H-2a, *J* = 17.0 Hz) 2.22 *d* (1H, H-2b, *J* = 17.0 Hz) 5.90 *tt* (1H, H-4, *J* = 6.9 1.2 Hz) 5.91 *d* (1H, H-7, *J* = 15.9 Hz) 6.82 *d* (1H, H-8, *J* = 15.9 Hz) 1.89 br*s* (3H, H-10 CH_3_) 1.07 *s* (3H, H-11 CH_3_) 1.03 *s* (3H, H-12 CH_3_) 1.90 *d* (3H, H-13 CH_3_ 1.2 Hz) 5.63 *t* (1H, H-14, *J* = 7.0 Hz) 4.51 *dd* (1H, H-15a, *J* = 12.3 7.6 Hz) 4.38 *dd* (1H, H-15b, *J* = 12.3 5.9 Hz) 4.28 *d* (1H, H-1′) 3.17 *t* (1H, H-2′, *J* = 8.0 Hz) 3.27–3.35 *m* (4H, H-3′, 4′, 5′) 3.88 *dd* (1H, H-6a′, *J* = 12.0, 1.7) 3.68 (1H, H-6b′, *J* = 12.0, 5.7 Hz).

^13^C-NMR (50.3 MHz CD4O) δ_C_: 42.7 (C-1) 49.6 (C-2) 200.0 (C-3 –C=O) 127.4 (C-4) 166.9 (C-5) 80.0 (C-6) 132.0 (C-7) 127.7 (C-8) 136.0 (C-9) 19.4 (C-10) 23.4 (C-11) 24.5 (C-12) 20.4 (C-13) 127.0 (C-14) 65.0 (C-15) 103.0 (C-1′) 74.6 (C-2′) 77.7 (C-3′) 71.3 (C-4′) 77.8 (C-5′) 62.7 (C-6′).

*Compound* **17**: ^1^H-NMR (400 MHz, CD_4_O) δ_H_: 8.07 *d* (2H, H-2′,6′, *J* = 8.7 Hz) 6.90 *d* (2H, H-3′,5′, *J* = 8.7 Hz) 6.34 *d* (1H, H-8, *J* = 2.1 Hz) 6.16 *d* (1H, H-6, *J* = 2.1 Hz) 5.06 *d* (1H, H-1′′, *J* = 7.8 Hz) 3.44 *dd* (1H, H-2′, *J* = 7.9, 7.5) 3.43–3.32 m* (3H, H-3′,4′,5′), 3.88 *brd* (1H, H-6a′, *J* = 11.0, 1.7 Hz) 3.64 *dd* (1H, H-6b′, *J* = 11.0, 5.9 Hz) 4.52 *d* (1H, H-1′′′ 1.7 Hz) 3.31* (1H, H-2′′′) 3.68 *m* (2H, H-3′′′, H-5′′′), 3.34* (1H H-4′′′)* 1.27 *d* (3H H-6′′′, *J* = 6.2 Hz).

^13^C-NMR (50.3 MHz CD_4_O)δ_C_: 159.5 (C-2) 136.1(C-3) 180.2 (C-4) 164.0 (C-5) 100.8 (C-6) 167.1 (C-7) 94.9 (C-8) 159.2 (C-9) 107.5 (C-10) 123.6 (C-1′) 132.2 (C-2′, 6′) 117.0 (C-3′, 5′) 162.2 (C-4′) 101.7 (C-1′′) 76.7 (C-2′′) 78.9 (C-3′′) 72.0^a^ (C-4′′) 78.0 (C-5′′) 69.3 (C-6′′) 102.9 (C-1′′′) 72.9^a^ (C-2′′′) 73.1 (C-3′′′) 74.3 (C-4′′′) 70.5 (C-5′′′) 18.6 (C-6′′′).

*: overlapped signals; ^a^: interchangeable signals.

*Compound* **18**: ^1^H-NMR (400 MHz, CD_4_O) δ_H_: 8.04 *d* (2H, H-2′,6′, *J* = 8.7 Hz) 6.82 *d* (2H, H-3′,5′, *J* = 8.7 Hz) 6.33 *d* (1H, H-8, *J* = 2.1 Hz) 6.22 *d* (1H, H-6, *J* = 2.1 Hz) 3.94 (3H, –OCH_3_) 5.84 *d* (1H, H-1′′, *J* = 7.8 Hz) 3.24 *dd* (1H, H-2′′, *J* = 8.5, 7.9) 3.43–3.32 *m*^a^ (3H, H-3′′,4′′,5′′), 3.99 *dd* (1H, H-6a′′, *J* = 11.7, 1.7 Hz) 3.64 *dd* (1H, H-6b′′, *J* = 11.3, 5.9 Hz). 4.51 *d* (1H, H-1′′′, *J* = 1.7 Hz) 3.86 *dd* (1H, H-2′′′, *J* = 3.1, 1.7 Hz) 3.68 *m* (3H, H-3′′′, *J* = 9.5 3.1 Hz), 3.34^a^ (1H H-4′′′) 3.67 (1H H-5′′′) 1.27 *d* (3H H-6′′′, *J* = 6.2 Hz).

^13^C-NMR (50.3 MHz CD_4_O) δ_C_: 128.8* (C-2′,6′) 128.8 (C-3′,5′) 128.3* (C-4′) 71.4 (C-7)103.0 (C-1′′) 75.0 (C-2′′) 77.9^b^ (C-3′′) 71.3 (C-4′′) 77.4^b^ (C-5′′) 67.6 (C-6′′) 102.1 (C-1′′′) 72.2(C-2′′′) 71.1^α^ (C-3′′′) 73.7 (C-4′′′) 71.1^α^ (C-5′′′) 17.9 (C-6′′′).

^a^,^b^: overlapped signals; *: interchanged signals.

*Compound* **19**: δ(ppm) 7.98 *d* (2H, H-2′, H-6′, *J* = 8.8 Hz), 7.44 *d* (1H, H-7′′′, *J* = 15.6 Hz), 7.36 *d* (2H, H-2′′′& H-6′′′, *J* = 9.2 Hz), 6.77 *d* (2H, d, H-3′ & H-5′, *J* = 8.8 Hz), 6.77 *d* (2H, H-3′′′ & H-5′′′, *J* = 9.2 Hz), 6.37 *d* (1H, H-8, *J* = 2.1 Hz), 6.14 *d* (1H, H-6 *J* = 2.1 Hz), 6.07 *d* (1H, H-8′′′, *J* = 15.6 Hz), 5.20 *d* (1H, H-1′′, *J* = 7.6 Hz), 4.35 *dd* (1H, H-6′′a, *J* = 12.6, 2.0 Hz), 4.21 *dd* (1H, H-6′′b, *J* = 12.8, 6.8 Hz), 3.35–3.51 *m* (4H, H-2″,3″,4′′,5′′) 159.5 (C-2) 137.0 (C-3) 179.4 (C-4) 162.7 (C-5) 99.7 (C-6) 165.8 (C-7) 94.9 (C-8) 158.3 (C-9) 105.7 (C-10) 123.4 (C-1′) 132.2 (C-2′,6′) 118.0 (C-3′,5′) 161.4 (C-4′) 104.1 (C-1′′) 74.9 (C-2′′) 77.9 (C-3′′) 71.8 (C-4′′) 75.2 (C-5′′) 64.5 (C-6′′) 123.2 (C-1′′′) 131.5 (C-2′′′ ,6′′′) 117.2 (C-3′′′ ,5′′′) 157.5 (C-4′′′) 147.5 (C-7′′′) 115.9 (C-8′′′) 168.7 (C-9′′′).

*Compound* **20**: 7.53 *s* (2H, H-2,6); 6.42 *d* (1H, H-8, *J* = 2 Hz), 6.21 *d* (1H, H-6, *J* = 2 Hz), 5.47 *d* (1H, H-1′′, *J* = 8 Hz), 3.94 *s* (6H, OCH_3_ × 2 at C-3′ and at C-5′) 5.47 *d* (1H, H-1′′, 7.8 Hz) 3.42–3.20 *m* (3H, H-2′′,3′′,4′′,5′′), 3.74 *dd* (1H, H-6a′′ 12.0, 5.8 Hz) 3.58 *dd* (1H, H-6b′′ 12.0, 2.1 Hz).

^13^C-NMR (50.3 MHz CD_4_O) δ_C_: 107.4 (C-2′,6′) 99.6 (C-6) 94.4 (C-8) 56.5 (–OCH_3_) 102.8 (C-1′′) 76.2 (C-2′′) 78.1^a^ (C-3′′) 71.0 (C-4′′) 77.3^a^ (C-5′′) 62.2 (C-6′′).

^a^: overlapped signals.

## Figures and Tables

**Table 1:** In vitro reducing ability (RA %) in DPPH assay, soybean lipoxygenase inhibition (% LOX inhbt) and anti-lipid peroxidation activity (A-LP %).

Compound	RA ^#^ % ± SD, DPPH, (20 min)	RA ^#^ % ± SD, DPPH, (60 min)	% LOX ± SD Inhbt @ (0.1 mM)	A-LP % ± SD @ (0.1 mM)
**1**	84 ± 1.8 *	100 ± 2.1 **	no	61 ± 0.6 **
**4**	86 ± 2.2 **	100 ± 3.1 **	no	13 ± 0.3 *
**5**	5 ± 0.1 *	no	29 ± 1.1 **	9 ± 0.1 *
**6**	8 ± 0.3 **	no	no	9 ± 0.1 *
**7**	2 ± 0.0 *	no	no	no
**8**	9.8 ± 0.4 *	no	no	7 ± 0.1 *
**9**	41 ± 1.0 **	42 ± 1.3 **	no	31 ± 0.7 *
**10**	24 ± 0.8 **	no	8.5 ± 0.1 **	18 ± 0.6 **
**11**	no	no	no	6 ± 0.1 *
**12**	no	no	no	16 ± 0.1 *
**13**	76 ± 1.1 **	87 ± 1.9 **	18 ± 0.6 **	53 ± 1.2 **
**14**	95 ± 3.2 **	100 ± 2.1 **	100 ± 2.5 **	100 ± 1.4 **
**15**	no	no	no	25 ± 1.0 *
**16**	7 ± 0.1 *	6 ± 0.0 *	25±1.2 **	45 ± 0.9 **
**17**	nt ^#^	nt ^#^	46 ± 1.0 **	nt ^#^
**18**	nt ^#^	nt ^#^	no	no
**19**	no	no	8 ± 0.3 *	no
**20**	2 ± 0.0 *	2 ± 0.0 *	no	no
**MeOH extract**	94 ± 1.9 **	100 ± 2.5 **	43 ± 0.4 *	57 ± 1.0 **
**MeOH:H_2_O (5:1) extract**	87 ± 2.1 **	8 ± 1.8 **	52 ± 0.7 **	no
**NDGA**	87 ± 1.1**	93 ± 1.8 **	91 ± 2.3 **	
**trolox**				88 ± 0.9 **

^#^ Final concentration 0.1mM; no: no activity under the experimental conditions; * *p* < 0.05; ** *p* < 0.01; nt ^#^: not tested (The amount of the compounds was very small for the experiments to be performed. Thus, we decided for these compounds to test only their enzyme inhibitory activity for the sake of comparison); significant differences are relative to the solvent control.

## Supplementary Materials

The following are available online at www.mdpi.com/2223-7747/7/1/1/s1.
