# Supplementary material for: The Occurrence of Flavonoids and Related Compounds in Cedrus brevifolia A. Henry ex Elwes & A. Henry Needles. Inhibitory Potencies on Lipoxygenase, Linoleic Acid Lipid Peroxidation and Antioxidant Activity
Source: Plants (Basel). 2017 Dec 27;7(1):1. doi: 10.3390/plants7010001 (PMC5874590; doi:10.3390/plants7010001)
Supplement: Supplementary file 1 [file plants-07-00001-s001.pdf]

^{1}H NMR spectra of the isolated compounds

The Occurrence of Flavonoids and Related Compounds in *Cedrus brevifolia* A. Henry ex Elwes & A. Henry Needles. Inhibitory Potencies on Lipoxygenase, Linoleic Acid Lipid Peroxidation and Antioxidant Activity

Andreas Douros [1], Dimitra Hadjipavlou-Litina [2], Konstantinos Nikolaou [3] and Helen Skaltsa [1,*]

Table of contentsPage

S1: ¹H-NMR (CD_3OD) spectrum of compound (**1**)taxifolin 2
S2: ¹H-NMR (CD_3OD) spectrum of compound (**2**) astragalin 3
S3: ¹H-NMR (CD_3OD) spectrum of compound (**3**) isorhamnetin 3-O-β-D-glucoside 4
S4: ¹H-NMR (CD_3OD) spectrum of compound (**4**)(-)-catechin 5
S5: ¹H-NMR (CD_3OD) spectrum of compound (**5**) benzoate glucoside 6
S6: ¹H-NMR (CD_3OD) spectrum of compound (**6**) benzyl-β-D-glucoside 7
S7: ¹H-NMR (CD_3OD) spectrum of compound (**7**) benzyl-β-D-rutinoside 8
S8: ¹H-NMR (CD_3OD) spectrum of compound (**8**)2-methoxy-phenyl-β-D-glucoside 9
S9: ¹H-NMR (CD_3OD) spectrum of compound (**9**) 3,4-dimethoxyphenyl-β-D-glucoside 10
S10: ¹H-NMR (CD_3OD) spectrum of compound (**10**) raspberry ketone 11
S11: ¹H-NMR (CD_3OD) spectrum of compound **(11)**p-anisic acid 12
S12: ¹H-NMR (CD_3OD) spectrum of compound (**12**) 4-hydroxybenzoic acid 4-O-β-D-glucoside 13
S13: ¹H-NMR (CD_3OD) spectrum of compound (**13)** p-coumaric acid and its glucoside 14
S14: ¹H-NMR (CD_3OD) spectrum of compound (**14**) p-coumaric acid glucoside 15
S15: ¹H-NMR (CD_3OD) spectrum of compound (**15**) *trans*-vaginoside 16
S16: ¹H-NMR (CD_3OD) spectrum of compound (**16**) abscisic alcohol glucoside 17
S17: ¹H-NMR (CD_3OD) spectrum of compound (**17**) kaempferol-3-*O*-β-rutinoside 18
S18: ¹H-NMR (CD_3OD) spectrum of compound (**18**)kaempferide-3-*O*-β-rutinoside 19
S19: ¹H-NMR (CD_3OD) spectrum of compound (**19**) tiliroside 20
S20: ¹H-NMR (CD_3OD) spectrum of compound (**20**) syringetin 3-O-β-D-glucoside 21
S21: Total weights of the isolated compounds 22

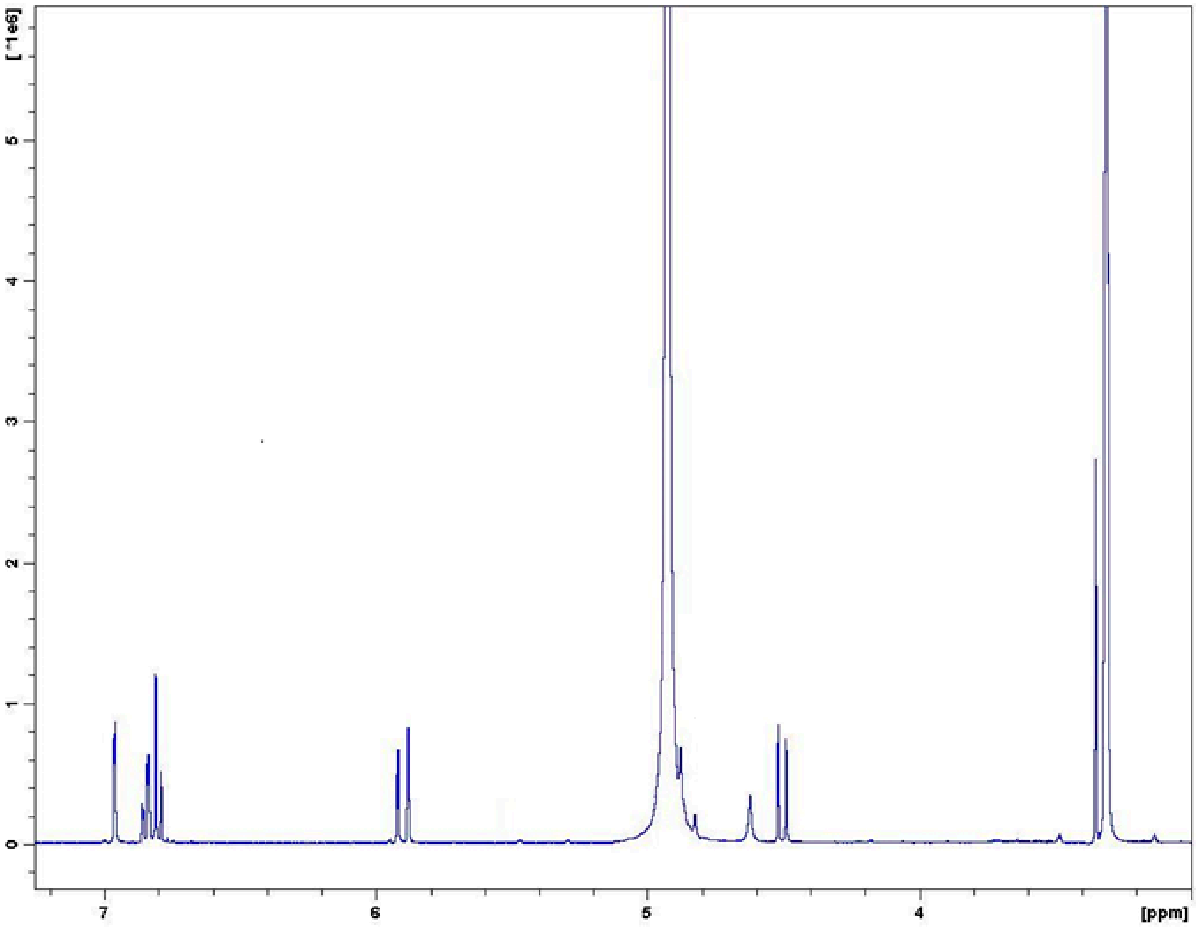

^{1}H –NMR spectrum of **1** (CD_3OD, 400 Hz)

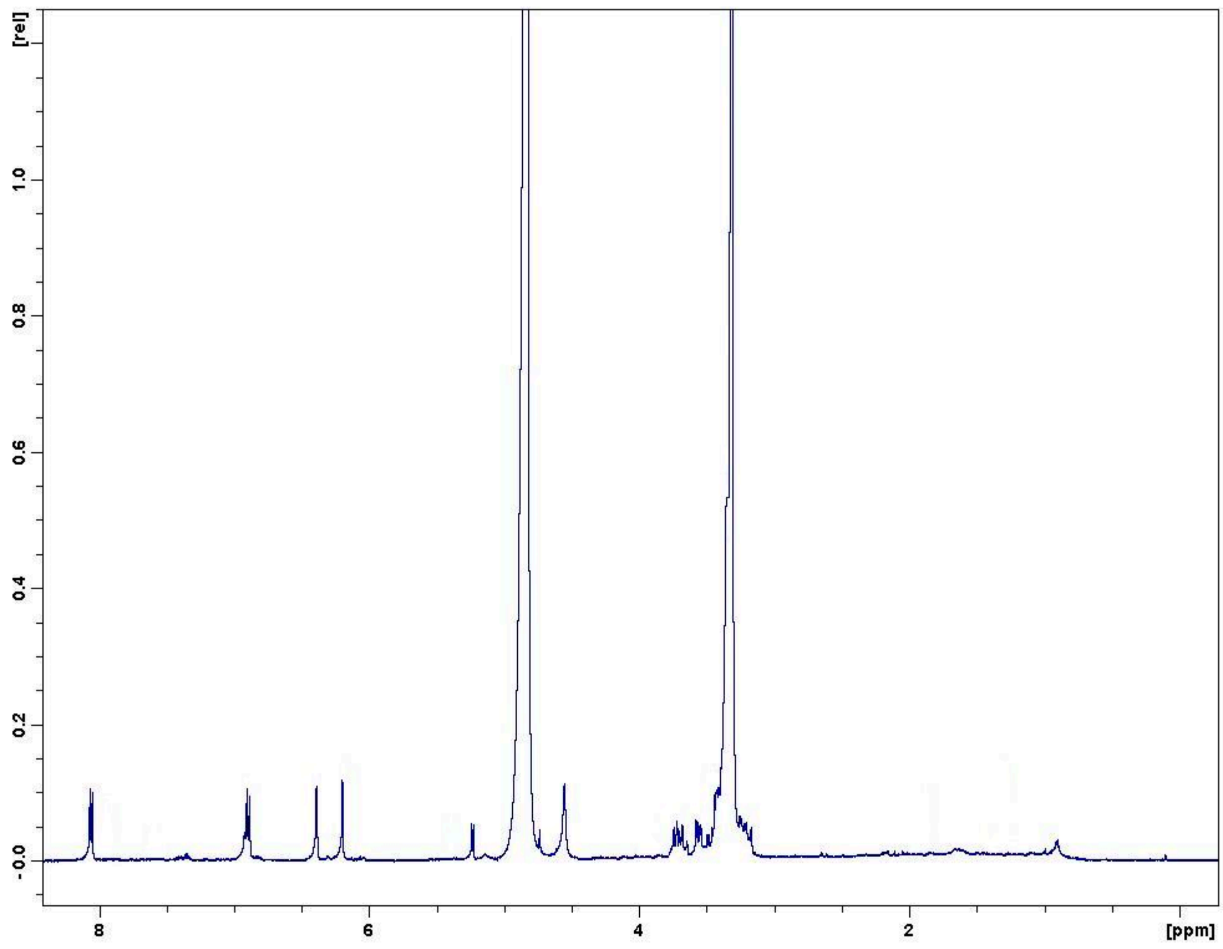

^1H –NMR spectrum of **2** (CD_3OD, 400 Hz)

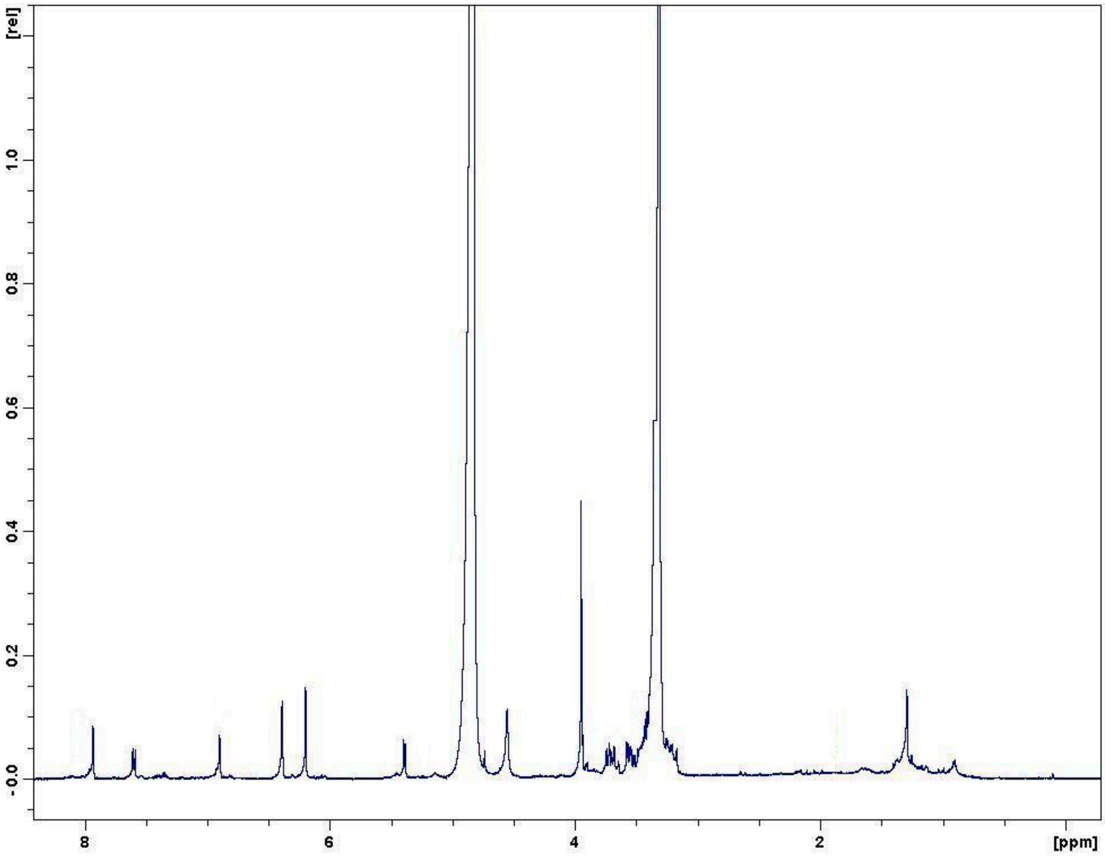

^{1}H –NMR spectrum of **3** (CD_3OD, 400 Hz)

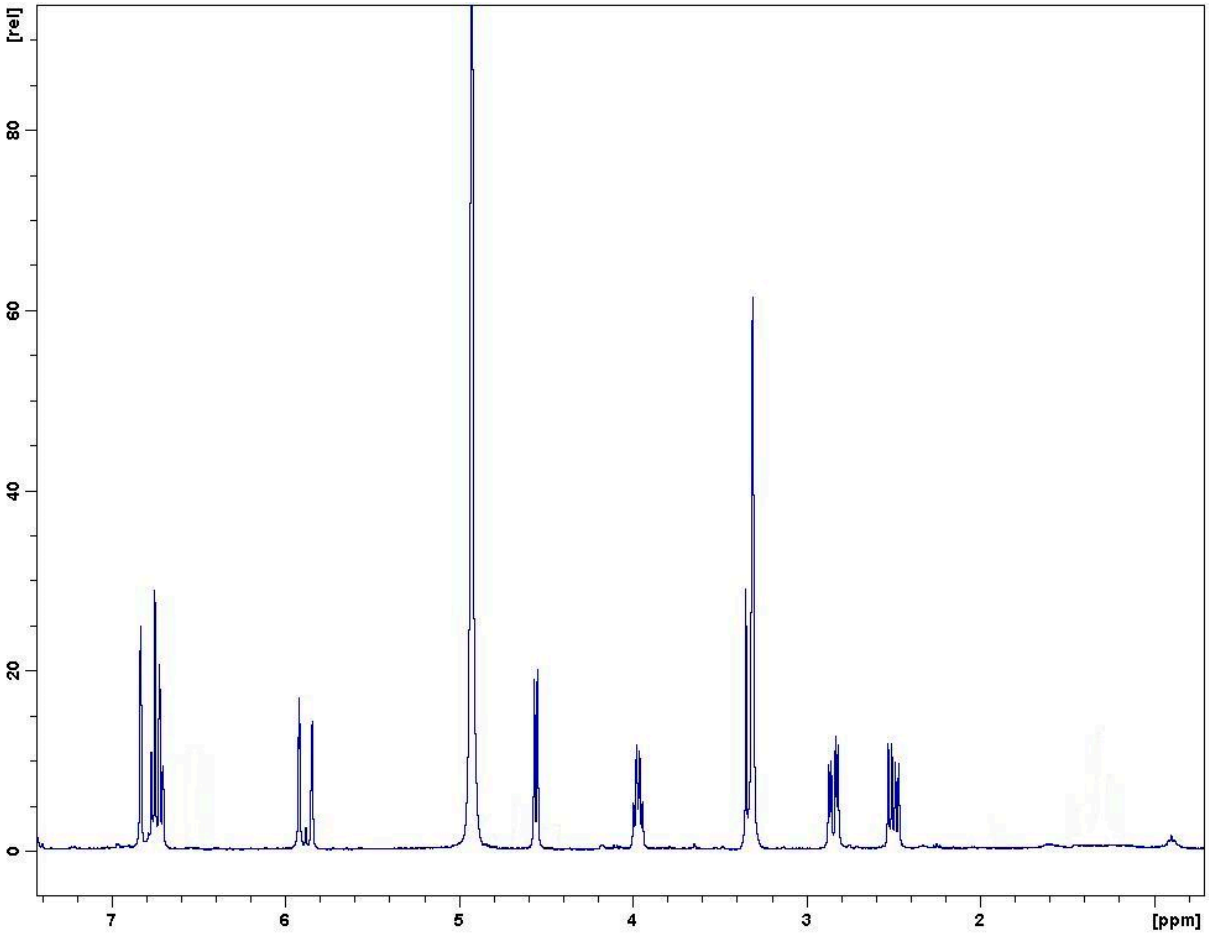

^{1}H –NMR spectrum of **4** (CD_3OD, 400 Hz)

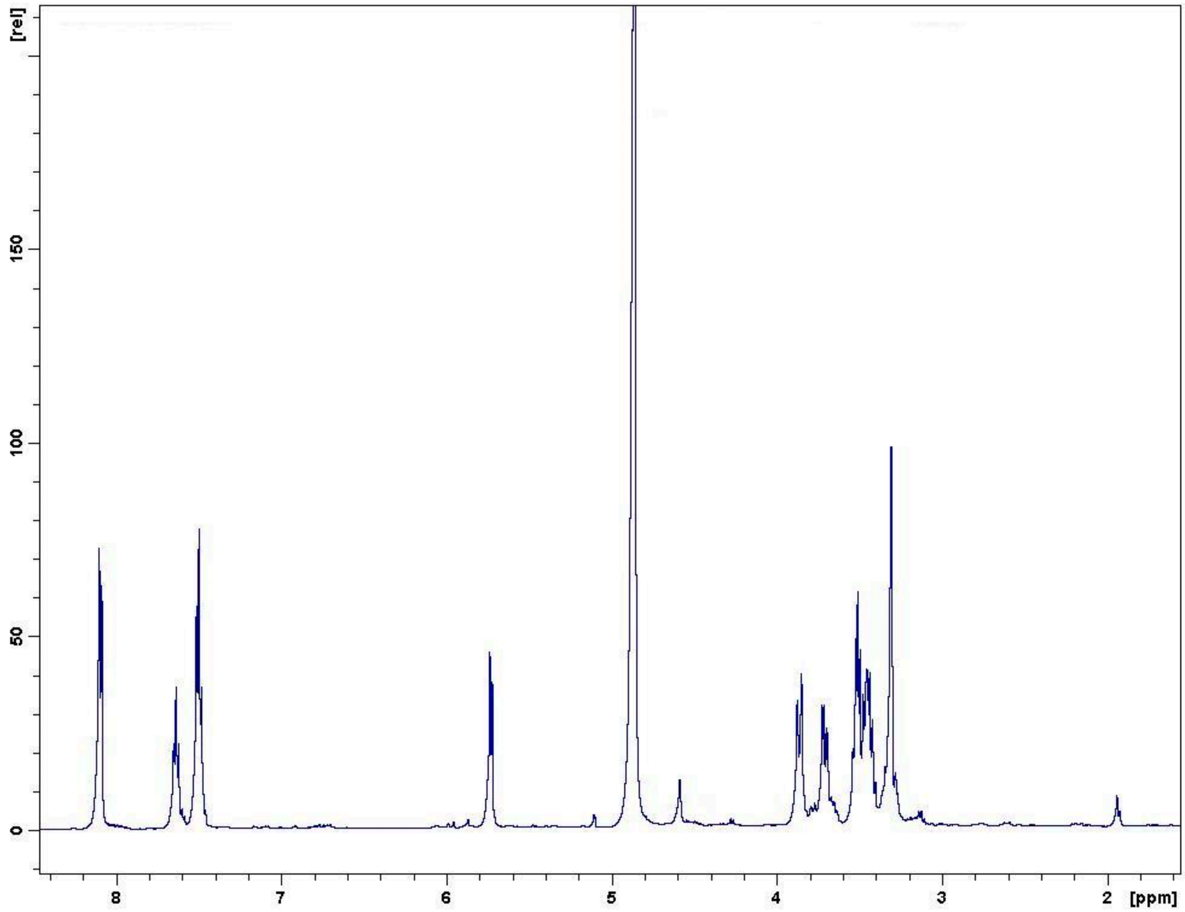

^{1}H –NMR spectrum of **5** (CD_3OD, 400 Hz)

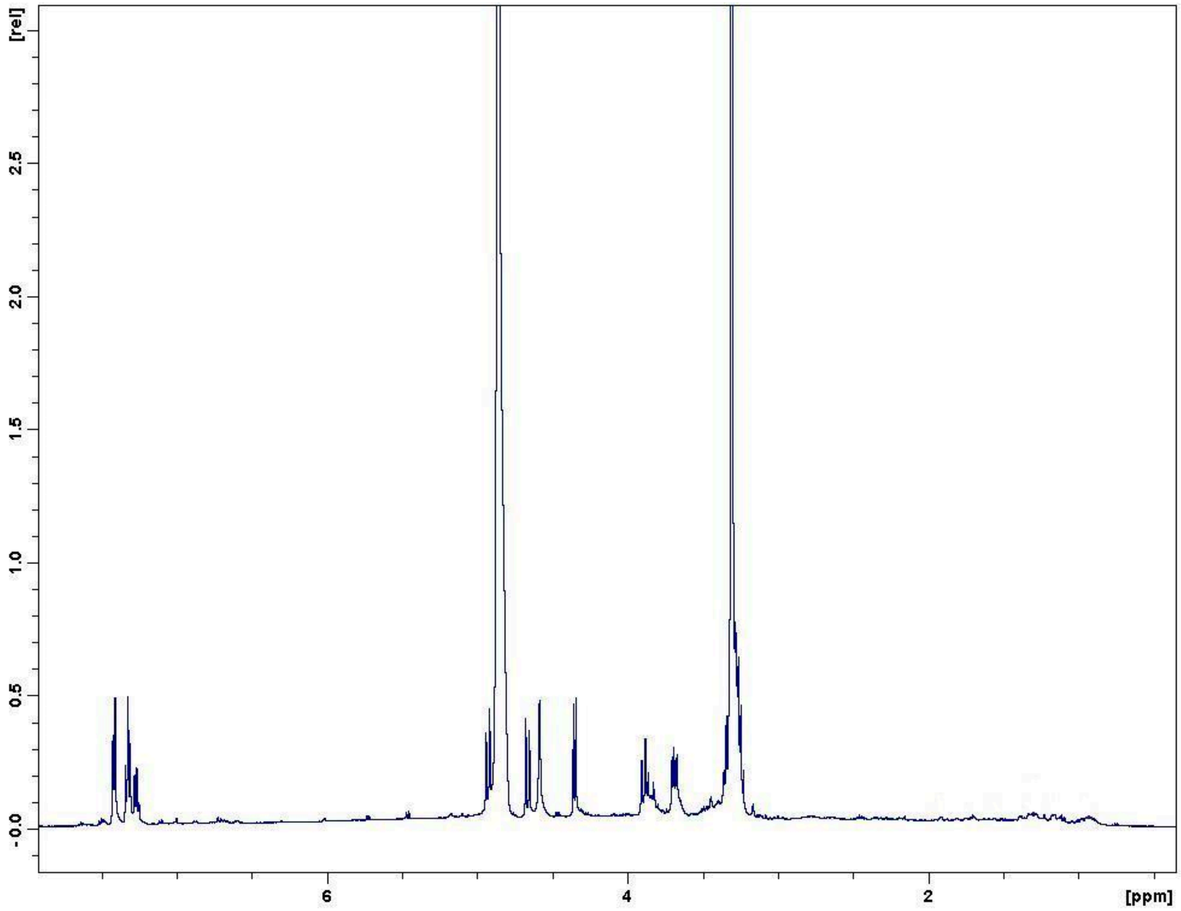

^{1}H –NMR spectrum of **6** (CD_3OD, 400 Hz)

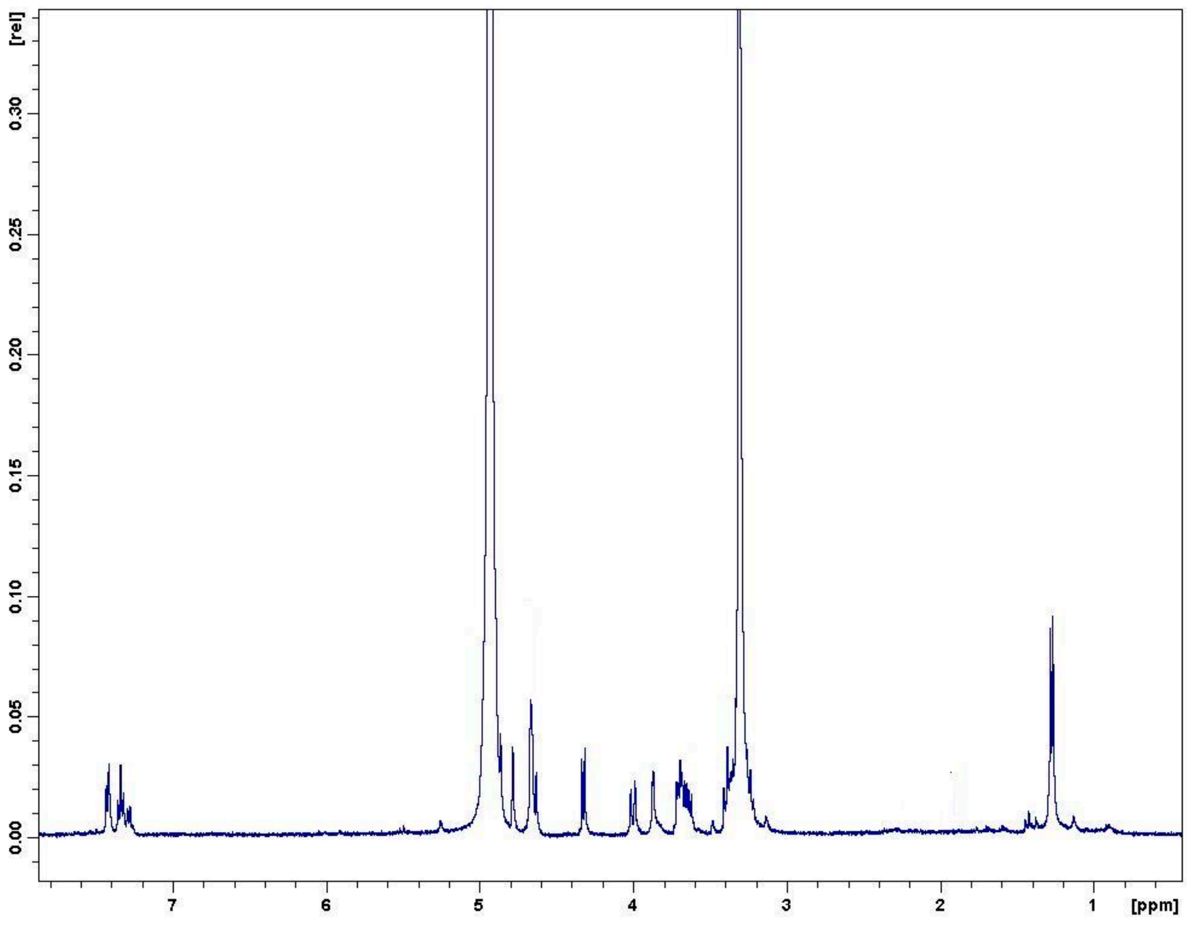

^{1}H –NMR spectrum of **7** (CD_3OD, 400 Hz)

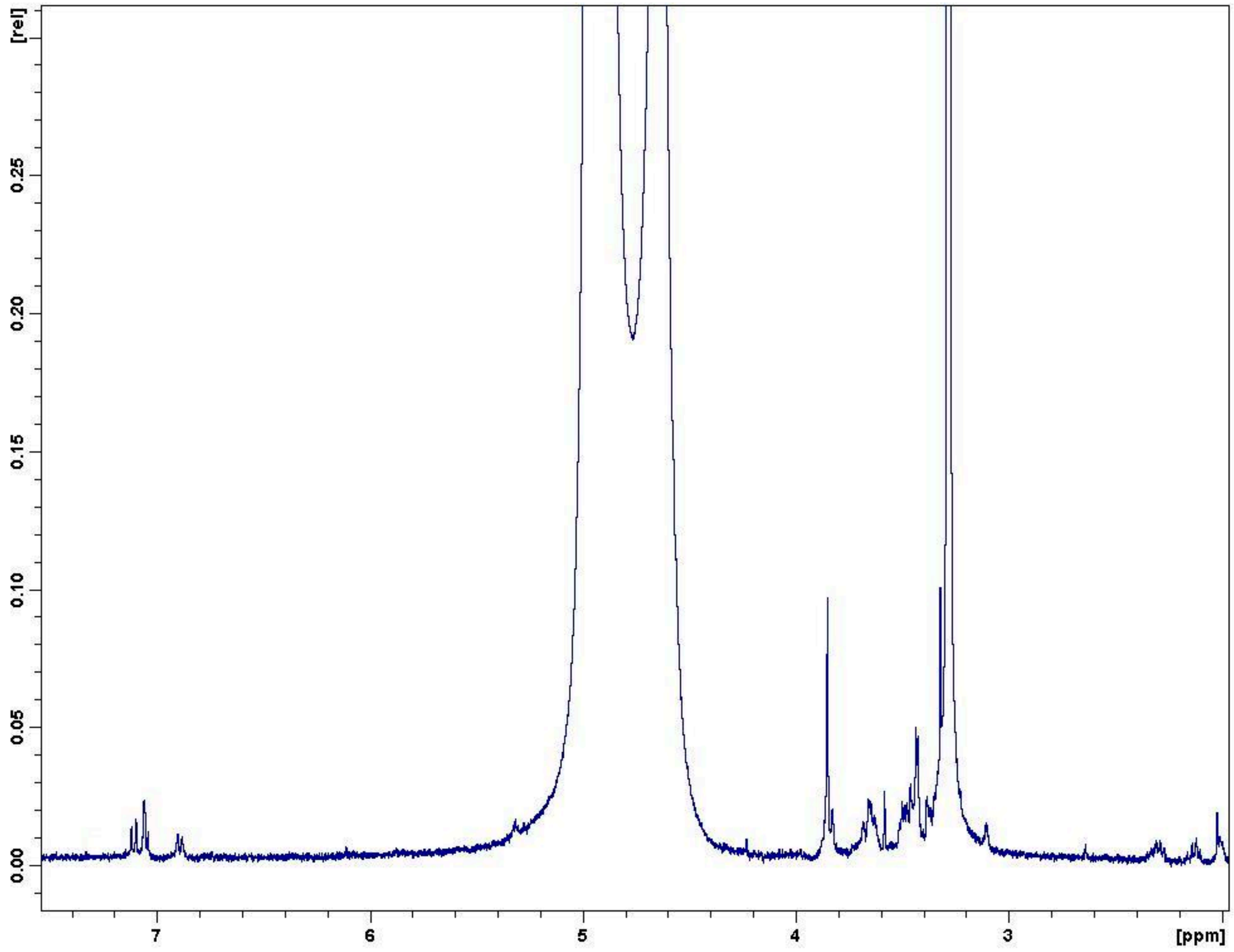

^{1}H –NMR spectrum of **8** (CD_3OD, 400 Hz)

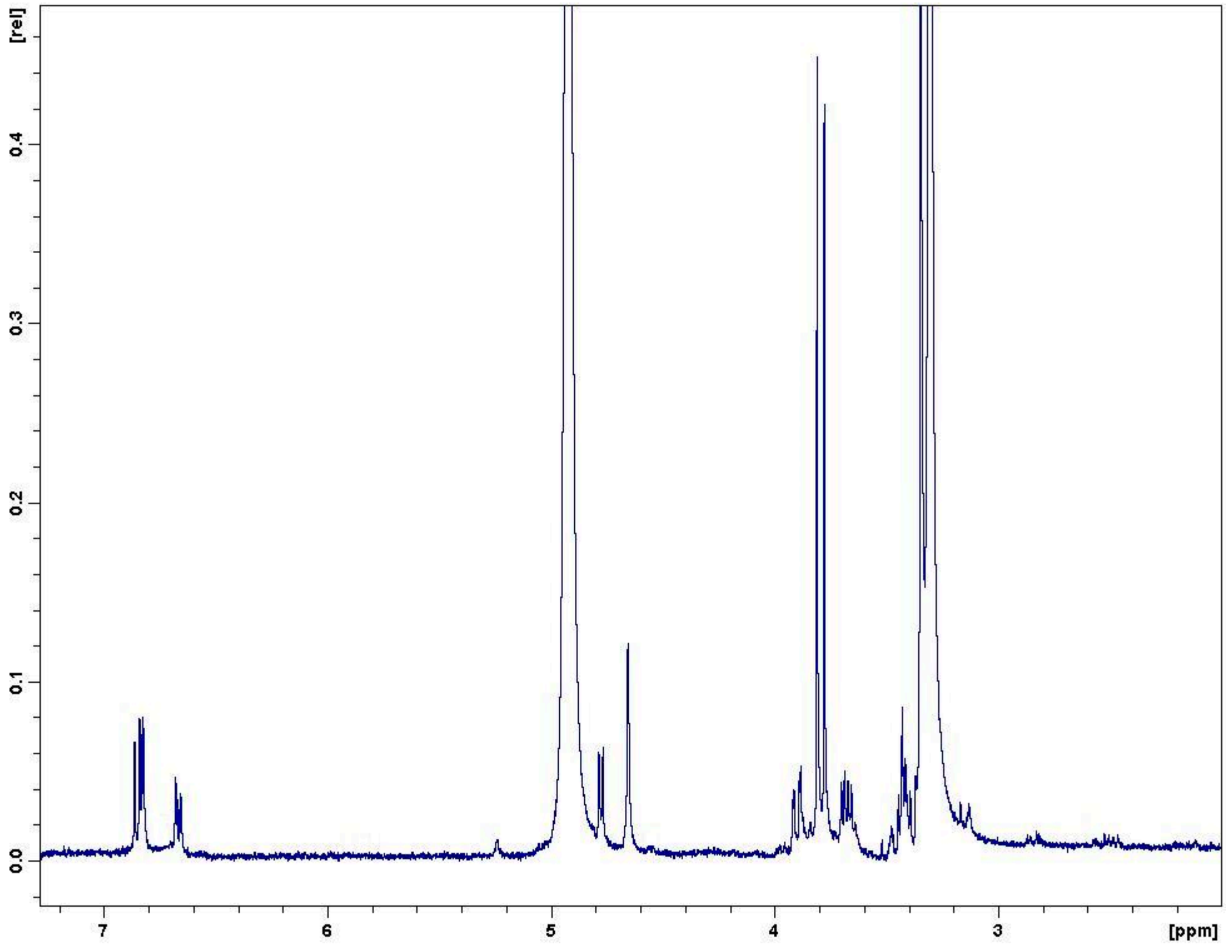

^{1}H –NMR spectrum of **9** (CD_3OD, 400 Hz)

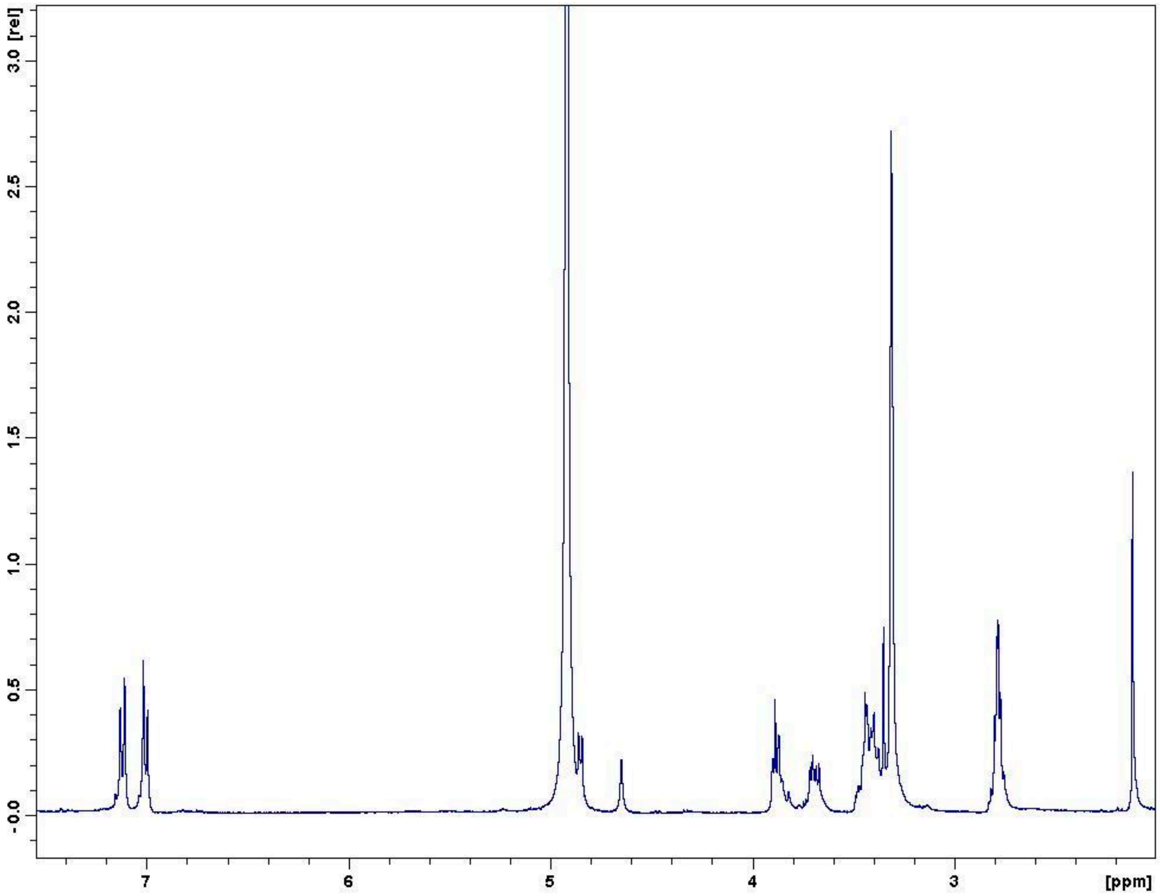

^{1}H –NMR spectrum of **10** (CD_3OD, 400 Hz)

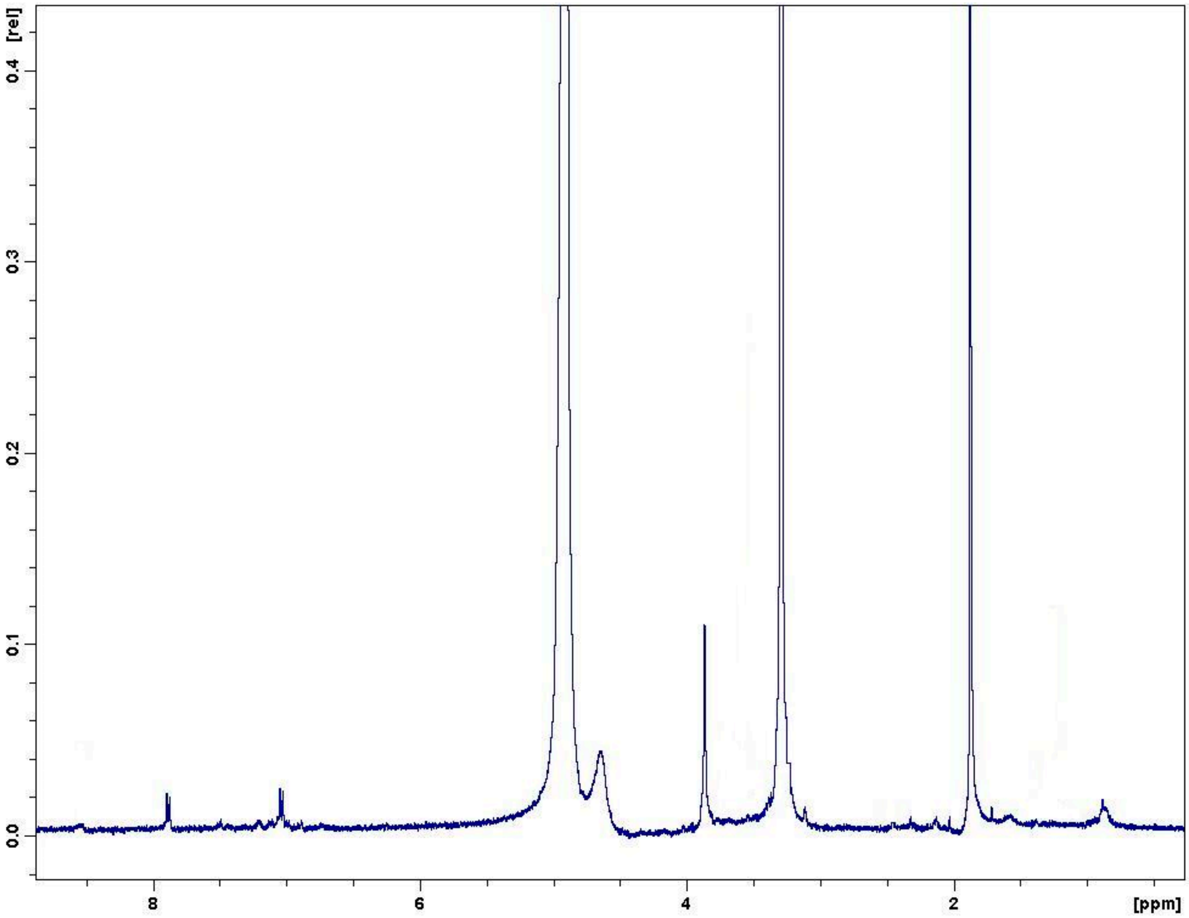

^{1}H –NMR spectrum of **11**(CD_3OD, 400 Hz)

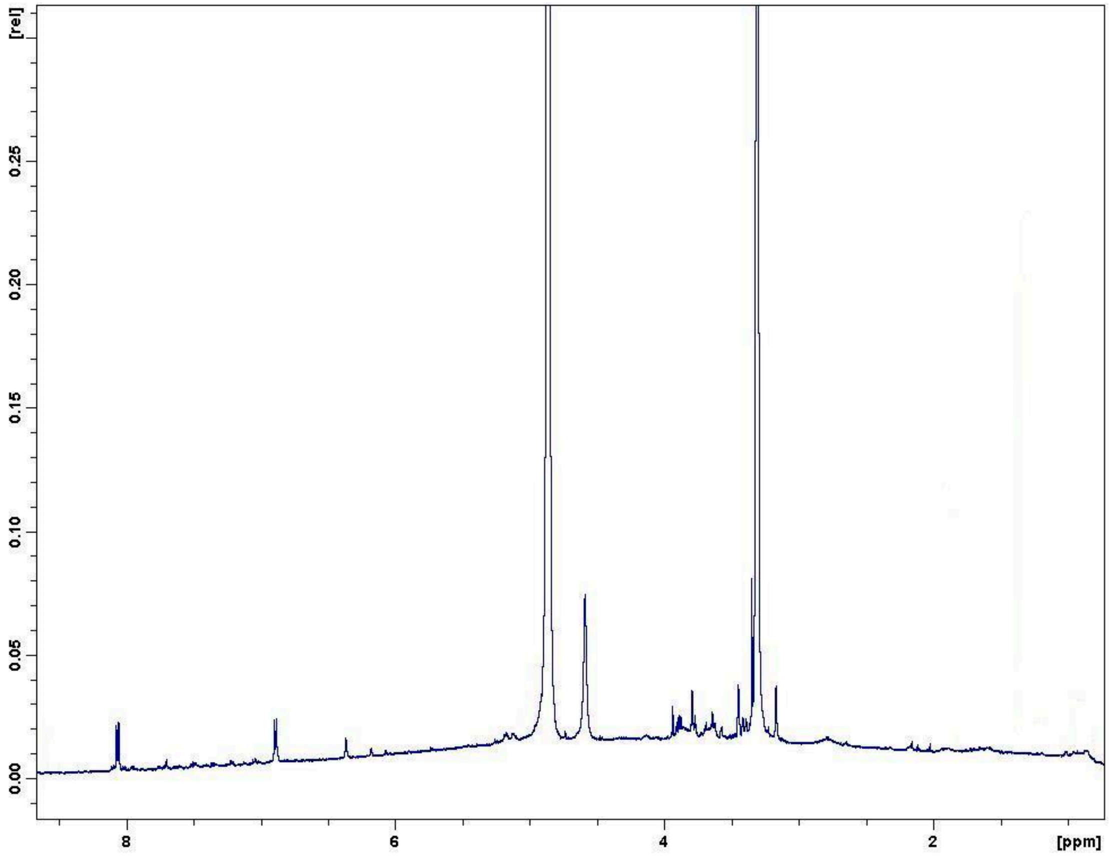

^1H –NMR spectrum of **12** (CD_3OD, 400 Hz)

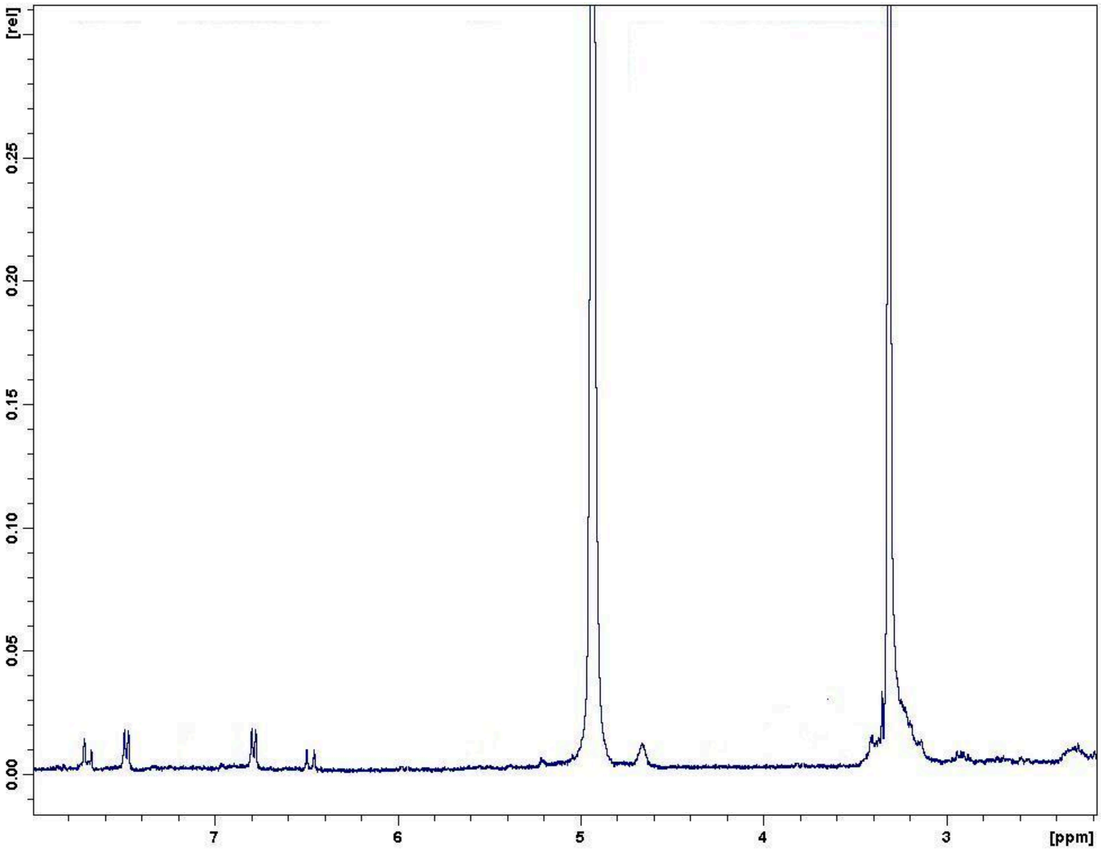

^{1}H –NMR spectrum of **13** (CD_3OD, 400 Hz)

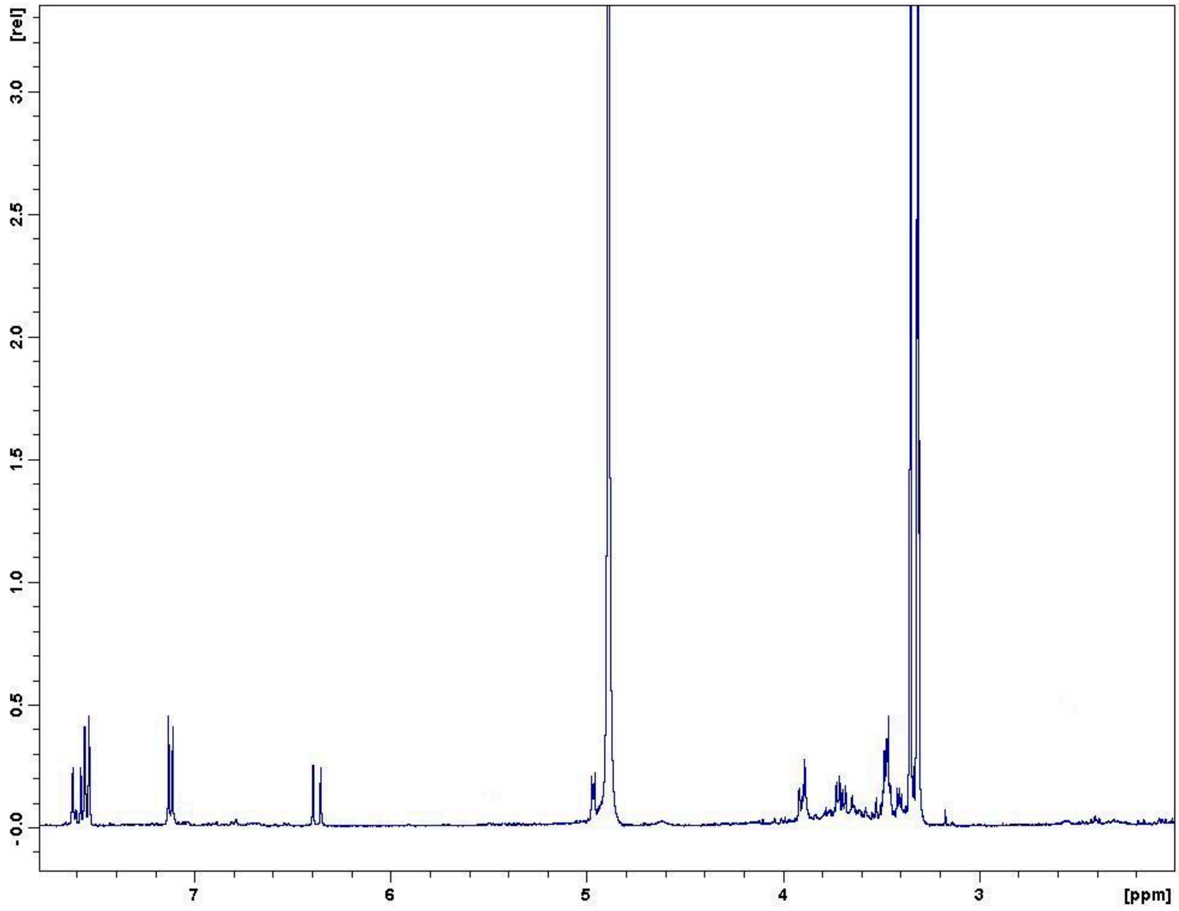

^{1}H –NMR spectrum of **14** (CD_3OD, 400 Hz)

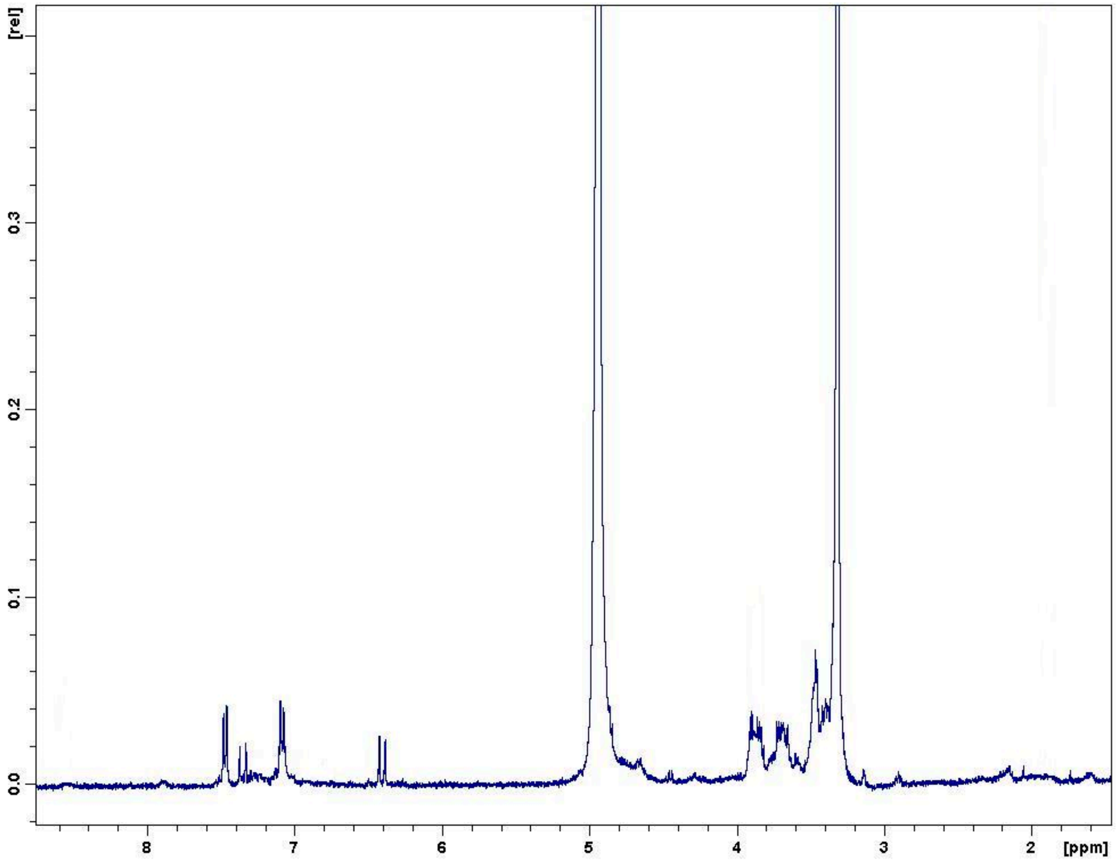

^{1}H –NMR spectrum of **15** (CD_3OD, 400 Hz)

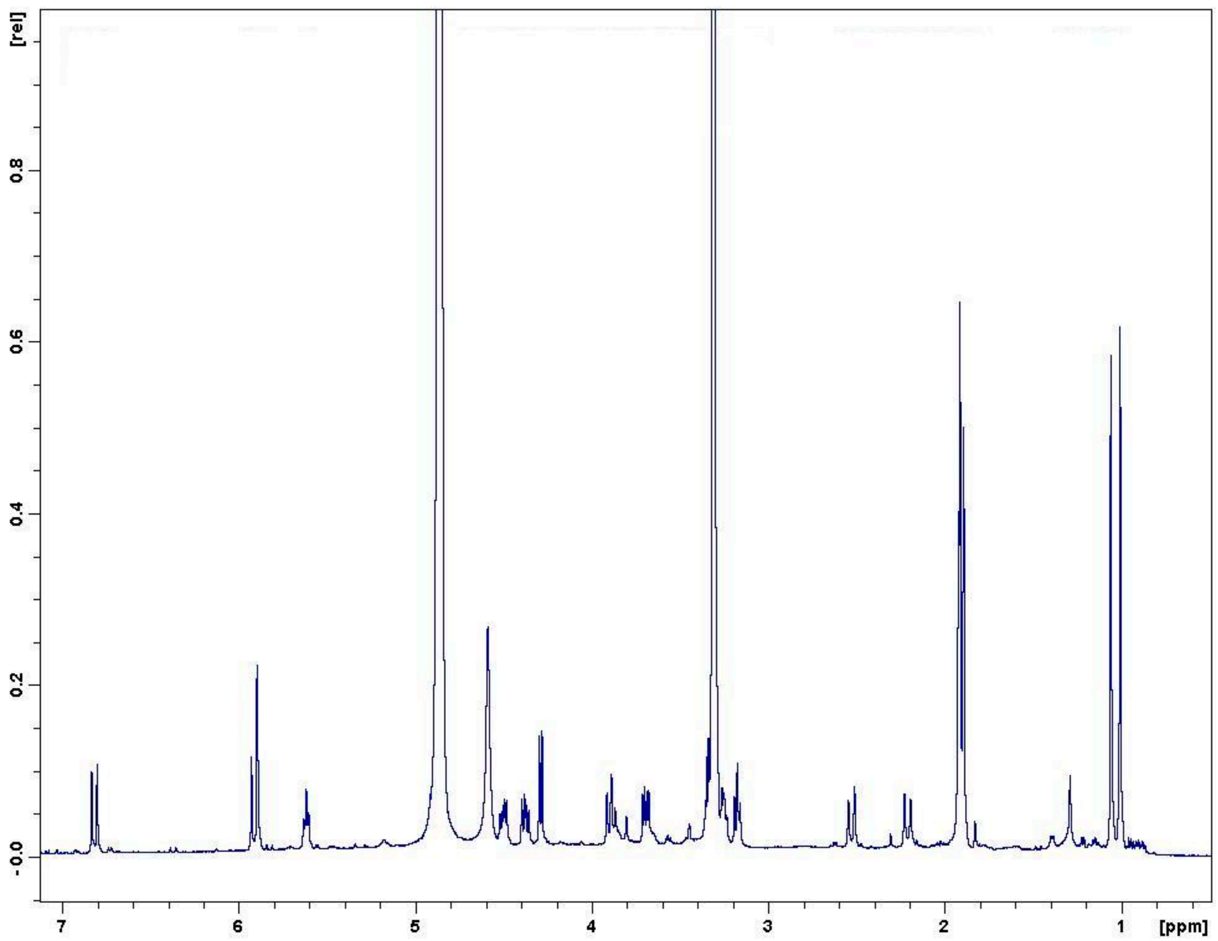

^{1}H –NMR spectrum of **16** (CD_3OD, 400 Hz)

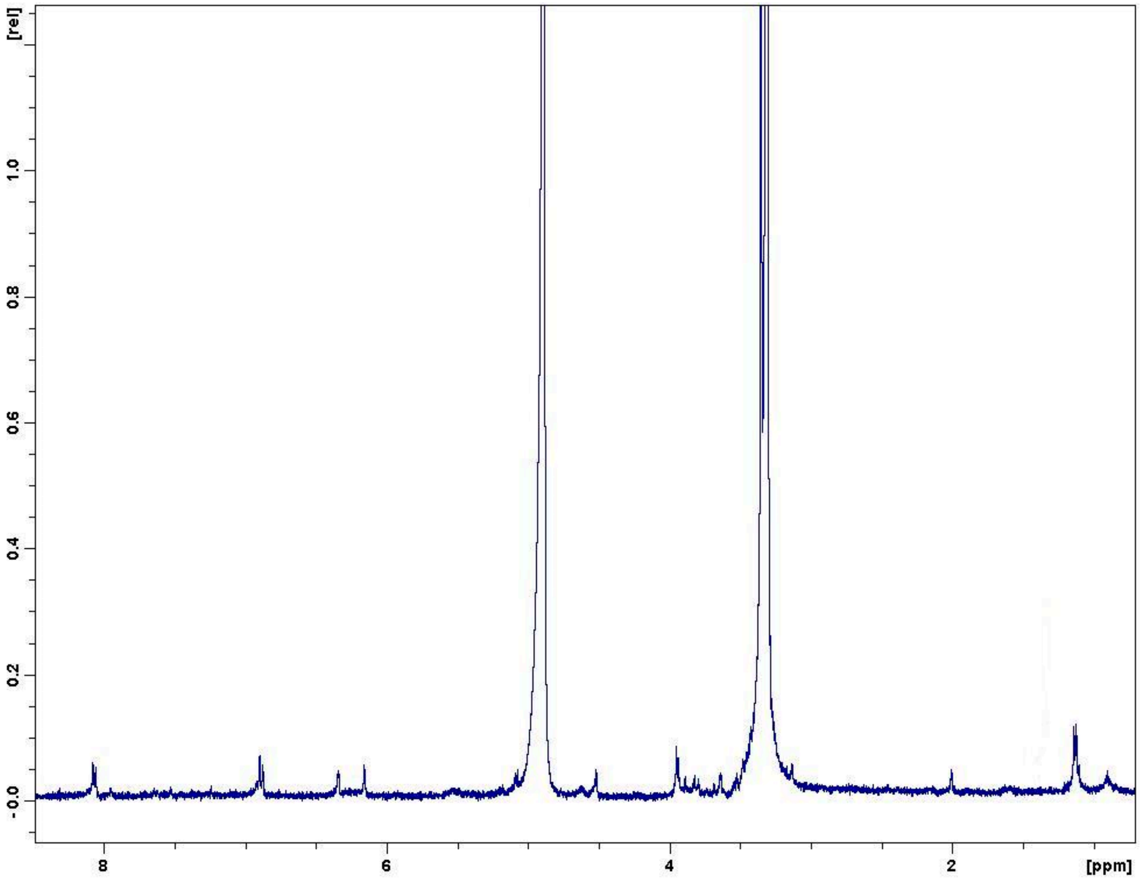

^{1}H –NMR spectrum of **17** (CD_3OD, 400 Hz)

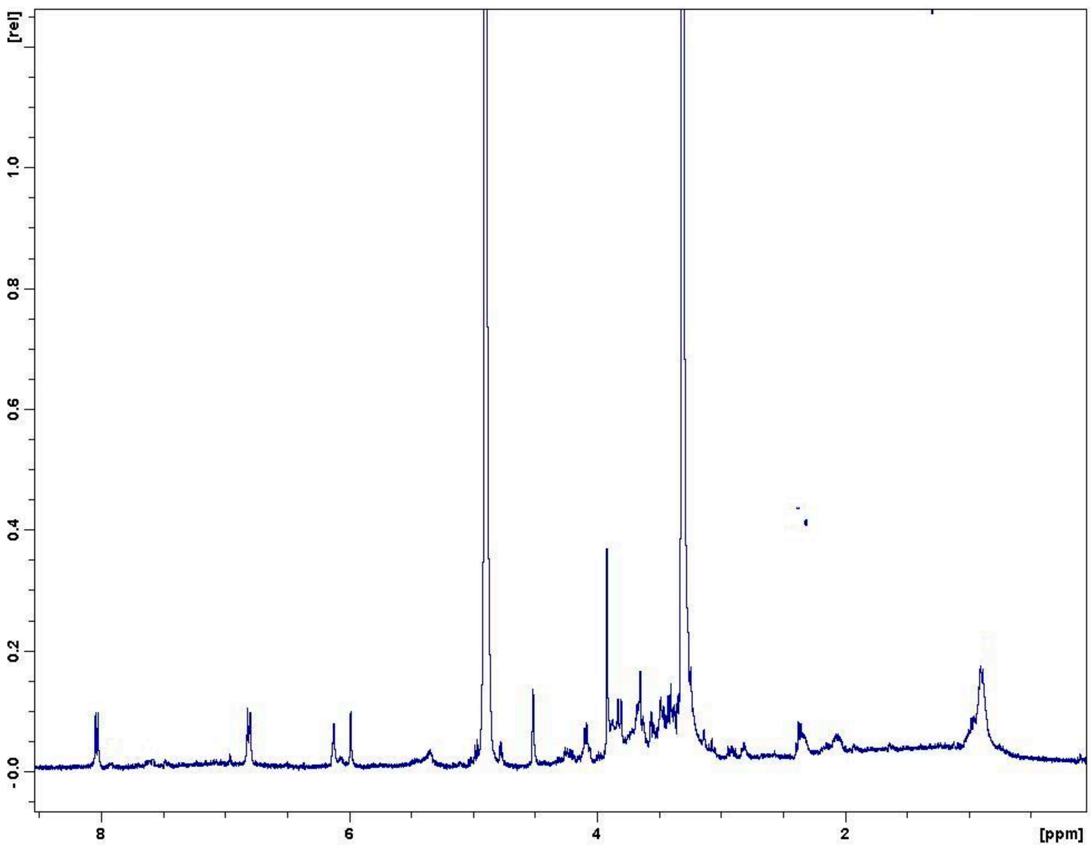

^{1}H –NMR spectrum of **18** (CD_3OD, 400 Hz)

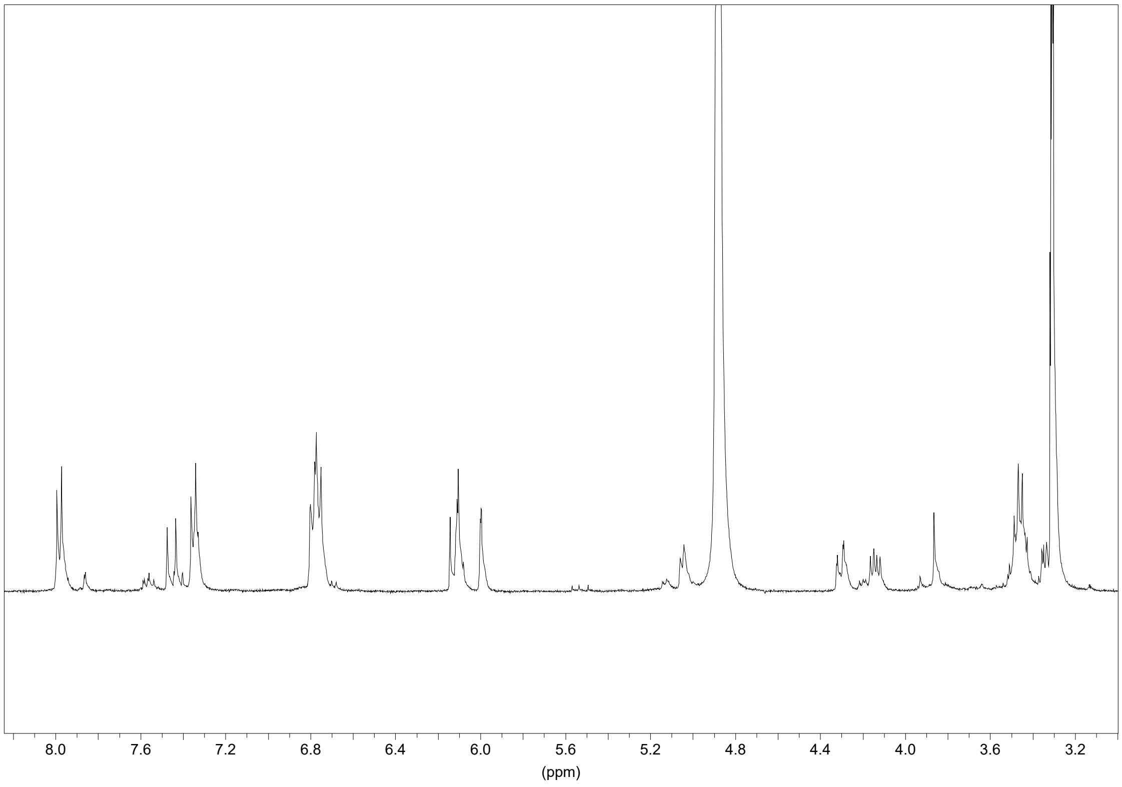

^{1}H –NMR spectrum of **19** (CD_3OD, 400 Hz)

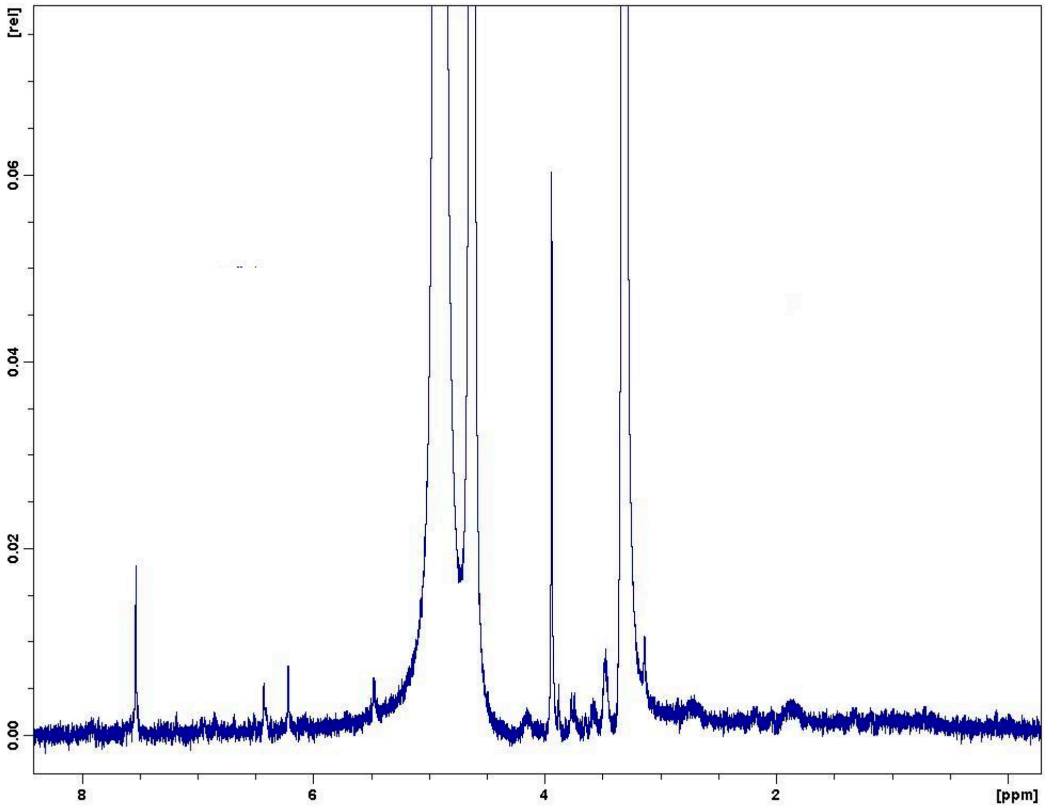

^{1}H –NMR spectrum of **20** (CD_3OD, 400 Hz)

Table S1. Total weights of the isolated compounds

Starting material	Fractions	Weights	Compound	Weight (mg)
**Methanol Extract 10.0 g**	I	1.5 g	**1**	9.1
	II	75.0 mg	**2**	0.4
			**3**	0.5
			**5**	12.6
			**6**	3.8
			**9**	2.7
			**10**	8.4
			**16**	3.4
			**12**	1.9
	IJ	358.7 mg	**4**	19.0
	IL	259.6 mg		14.5
	IK	142.0 mg		7.5
	IJ	358.7 mg	**7**	2.8
	D'	149.6 mg	**8**	4.0
			**13**	6.0
	H	236.7 mg	**11**	3.0
			**15**	2.8
	IL	259.6 mg	**14**	3.5
**Methanol: Water (5:1) Extract 5.0 g**	I	157.0 mg	**17**	1.0
			**18**	2.8
			**19**	5.7
			**20**	0.8
